# Origin of dielectric polarization suppression in confined water from first principles†

**DOI:** 10.1039/d3sc04740g

**Published:** 2023-12-04

**Authors:** T. Dufils, C. Schran, J. Chen, A. K. Geim, L. Fumagalli, A. Michaelides

**Affiliations:** a Department of Physics and Astronomy, University of Manchester Manchester M13 9PL UK laura.fumagalli@manchester.ac.uk; b National Graphene Institute, University of Manchester Manchester M13 9PL UK; c Cavendish Laboratory, Department of Physics, University of Cambridge Cambridge CB3 0HE UK; d Lennard-Jones Centre, University of Cambridge Trinity Ln Cambridge CB2 1TN UK am452@cam.ac.uk; e School of Physics, Peking University Beijing 100871 China; f Yusuf Hamied Department of Chemistry, University of Cambridge Lensfield Road Cambridge CB2 1EW UK

## Abstract

It has long been known that the dielectric constant of confined water should be different from that in bulk. Recent experiments have shown that it is vanishingly small, however the origin of the phenomenon remains unclear. Here we used *ab initio* molecular dynamics simulations (AIMD) and AIMD-trained machine-learning potentials to understand water's structure and electronic properties underpinning this effect. For the graphene and hexagonal boron-nitride substrates considered, we find that it originates in the spontaneous anti-parallel alignment of the water dipoles in the first two water layers near the solid interface. The interfacial layers exhibit net ferroelectric ordering, resulting in an overall anti-ferroelectric arrangement of confined water. Together with constrained hydrogen-bonding orientations, this leads to much reduced out-of-plane polarization. Furthermore, we directly contrast AIMD and simple classical force-field simulations, revealing important differences. This work offers insight into a property of water that is critical in modulating surface forces, the electric-double-layer formation and molecular solvation, and shows a way to compute it.

## Abstract 2

Atomistic simulations explain recent experimental findings of dielectric polarization suppression in confined water, revealing it originates in an anti-ferroelectric order driven by spontaneous antiparallel alignment of water dipoles at surfaces.

## Graphical abstract

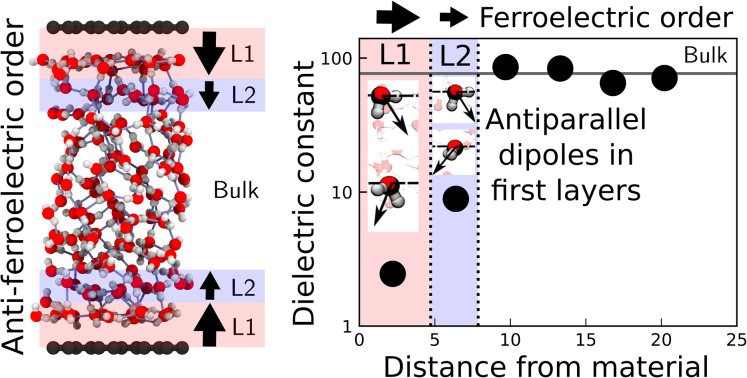

## Introduction

The reduction of the dielectric constant of interfacial and confined water has been the subject of extensive studies for many decades because of the ubiquitous presence of electrified surface/water interfaces in materials science, geology, chemistry and molecular biology as well as the development of new nanotechnologies (see *e.g.* ref. 1–8). However, a clear demonstration of this effect proved difficult due to challenges in both experimentally probing a dielectric response of only a few water layers near surfaces and in accurately computing it. Recently, the dielectric response of thin water layers confined in single slit-like nanochannels made of van der Waals crystals has been measured on the atomic scale.^9^ These measurements, which were conducted with nanoslits made of graphene and hexagonal boron nitride (hBN), revealed the presence of an electrically “dead” water layer near the slit walls with a surprisingly low dielectric constant in the direction perpendicular to the surface (*ε*_⊥_ ≈ 2) and extending around two molecular layers (∼7 Å) into the bulk.

The observation of an interfacial water layer with an out-of-plane dielectric constant approximately 1/40th that of bulk water sparked renewed interest in understanding the dielectric polarization properties of nanoconfined water. In particular, there has been a surge of theory and simulation work (see *e.g.* ref. 10–26), including a large number of force-field molecular dynamics (FFMD) with widely used point charge models. These studies have been of tremendous value and generally predict a decrease of *ε*_⊥_ for water near surfaces, in qualitative agreement with the recent experiments. However, the intensity of the decrease, how far it extends from the surface into the bulk, and the origin of the effect are all issues under debate. In addition, the FFMD studies involve the application of water force-fields parameterised to describe bulk rather than interfacial water properties,^27^ and the electronic coupling between water and the confining materials is not taken into account. Indeed, previous FFMD studies have mostly focused on water confined in graphene nanoslits. This means that it remains unclear whether the dielectric properties of nanoconfined water differ (if at all) between graphene and hBN. Such considerations call for the application of a first-principles based simulation approach such as density functional theory (DFT). DFT is not without its shortcomings, particularly when dealing with water. However, when a suitable choice of exchange–correlation functional is made it can deliver the requisite accuracy for water–surface interactions^28^ and when combined with molecular dynamics – so-called *ab initio* molecular dynamics (AIMD) – has been used successfully to probe the structure and dynamics of nanoconfined water (see *e.g.* ref. 13 and 29–31). However, DFT has yet to be used to deliver explicit estimates of the dielectric properties of confined water, mainly because of methodological challenges in extracting an accurate dielectric constant from computationally expensive AIMD simulations.

In this study, we exploit recent developments that enable the determination of dielectric properties with AIMD^32,33^ to calculate water's dielectric polarization inside graphene and hBN nanoslits. We analyze the molecular origin behind water's dielectric properties by calculating the magnitude and orientational distribution of the water molecule dipole moments as well as the topology of the hydrogen bonding network near the slit surfaces. This analysis is aided by the development of AIMD-trained machine learning (ML) neural network potentials (NNP) which ensure that converged structural properties of nanoconfined water are obtained. We show that our AIMD simulations describe the experimental results with fairly good accuracy, predicting a much reduced *ε*_⊥_ that originates in the first two interfacial water layers, where the water dipoles are constrained in an antiparallel configuration with respect to the normal to the surface in a ferroelectric configuration. This in turn leads to a reduced effective polarization in the perpendicular direction, irrespective of the distance between the confining surfaces and their electronic structure. We contrast our DFT-based MD simulations with those obtained from classical force-field simulations, in particular simple point charge water models used in previous studies. They reveal significant differences in the predicted arrangement of the water dipoles near the solid interface, indicating that the electronic properties of the surface are critical for quantitatively computing properties of interfacial and confined water.

## Results

**Fig. 1:**
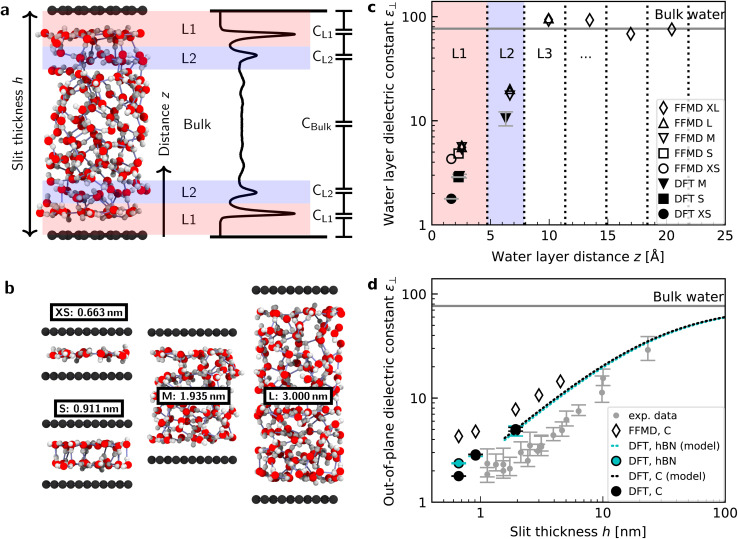
Dielectric constant of water confined between graphene and hBN. (a) Snapshot of a typical geometry for water confined within a nanoslit (left part of panel (a)) and the planar averaged water density profile (right part of panel (a)). Various terms used throughout the manuscript (*h*, *z*, L1, L2, and the capacitor model) are also indicated in this panel. (b) Snapshots for different confining distances and corresponding nomenclature (see details in ESI Table S1†). (c) Calculated *ε*_⊥_ for individual water layers as a function of the distance from graphene using *ab initio* (filled symbols) and force-field (open symbols) molecular dynamics calculations. Vertical dashed lines indicate the thickness of the water layers. Light red and blue colored areas highlight the first and second interfacial layer, L1 and L2, respectively. Dielectric constants obtained by FFMD calculations have been increased by 0.8 to account for the electronic polarizability. As shown by the presence of the error bars, the point for the dielectric constant of L1 for 1.935 nm [M] lies under the one for DFT and 0.911 nm [S]. (d) Effective *ε*_⊥_ as a function of the separation distance between two confining surfaces calculated using the capacitance model in eqn (7). Filled symbols are *ab initio* calculations for graphene (black) and hBN (cyan) slits. The dashed black (cyan) curve is the *ab initio* prediction using the capacitor model and the interfacial *ε*_⊥_ obtained from DFT calculations for graphene (hBN) at separation distance *h* = 1.935 nm [M]. Open symbols are force-field simulations for the graphene slit. Dielectric constants obtained by FFMD calculations have been increased by 0.8 to account for the electronic polarizability. Grey symbols are the experimental data from ref. 9, shifted by 3.354 Å in the *x*-axis direction according to the definition of *h* in this study (atom-to-atom distance of the slit, not water thickness).

We performed simulations with AIMD, AIMD-trained ML potentials, and FFMD for water confined within graphene and hBN nanoslits, for simplicity referred to as graphene and hBN slits, as described in Materials and methods. To study the effect of extreme confinement on interfacial water, as in the experiments, we analyzed various separation distances between the confining layers: from 0.66 to 2.00 nm in AIMD simulations, and from 0.66 to 4.5 nm in FFMD simulations (labelled XS [0.66 nm], S [0.91 nm], M [1.94 nm], L [3.00 nm] and XL [4.50 nm], following the nomenclature used in ref. 13, as shown in Fig. 1b, see details in ESI Table S1†). For each distance, we took care to establish the structure of nanoconfined water films, in agreement with previous work (see *e.g.* ref. 29 and 34–37) that report density oscillations in the water structure perpendicular to the surface of the confining layers. An example of such a density profile obtained from the AIMD-trained ML potential is shown in Fig. 1a, from which it can be seen that at least two clear solvation layers of water can be identified at each interface.

Having established the water structure, we calculated *ε*_⊥_ of water using the finite-field method.^38–40^ In this approach the dielectric displacement *D* is related to the local Maxwell field ***E*** and the local polarization ***P*** by the relation ***D***(*z*) = ***E***(*z*) + 4π***P***(*z*). In the slit geometry, in the absence of free charges, the dielectric displacement in the perpendicular direction, *D*_⊥_, is independent of *z*. Therefore it is advantageous to use it here as an independent electric variable. A variation in the local perpendicular component of the electric field, Δ*E*_⊥_, can then be obtained from the variation Δ*D*_⊥_ as

1 Δ*E*_⊥_ = ∫*ε*_nl_^−1^(*z*,*z*′)Δ*D*_⊥_(*z*′)d*z*′

where *ε*_nl_^−1^(*z*,*z*′) is the non-local response kernel, linking the variations of *E*_⊥_ at position *z* to the variations of *D*_⊥_ at position *z*′. Because *D*_⊥_ is independent of *z*, the local dielectric constant can be defined as *ε*_⊥_^−1^(*z*) = ∫*ε*_nl_^−1^(*z*,*z*′)d*z*′ and obtained from the local polarization response in the perpendicular direction Δ*P*_⊥_(*z*) = *P*_⊥,*D*_(*z*) − *P*_⊥,0_(*z*) as2
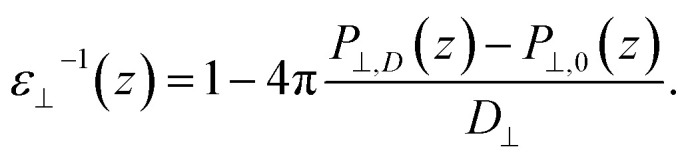


While in previous work^14^ the ratio 
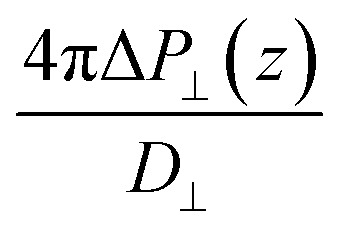
 was computed from the fluctuation–dissipation relation,^5^ here we computed it directly from the change of polarization Δ*P*_⊥_ upon a change in the dielectric displacement *D*_⊥_ using the finite-field method.^38–40^ This is similar to what was done in ref. 8 (see Materials and methods), and to do so, we applied a field of magnitude −*D*_⊥_, which leads to3
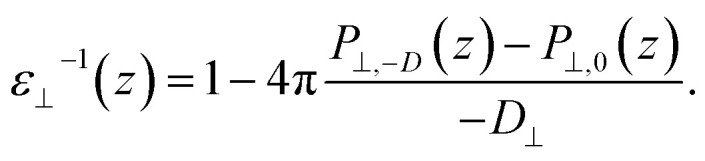


By combining eqn (2) and (3), we then obtained4
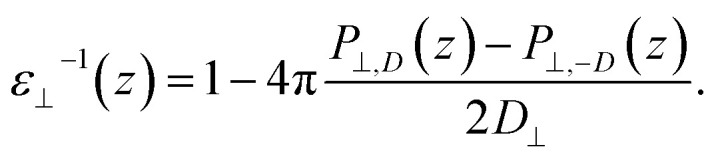


The advantage of this approach is that the fluctuations of *P*_⊥,*D*_(*z*) − *P*_⊥,0_(*z*) are of the same order of magnitude as the fluctuations of *P*_⊥,−*D*_(*z*) − *P*_⊥,0_(*z*). By using eqn (4), the fluctuations of the ratio of polarization variation over electric displacement is divided by 2, compared to the use of +*D*_⊥_ and 0. This allows the dielectric constant to converge faster, which is particularly important in the context of AIMD. We computed the local polarization *P*(*z*) from the charge density averaged over the parallel directions as5
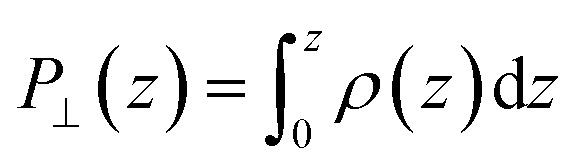
where we assumed that *P*_⊥_(0) = 0, since *z* = 0 in our setup is located in the vacuum far from any atoms. A validation of this assumption can be found in the ESI.† The obtained values of polarization were corrected by subtracting the local polarization response of the system in the absence of water, that is, composed of only the confining surfaces (see ESI Fig. S2†). This allowed us to extract the dielectric response of confined water and avoid the direct contribution of the surface, in analogy with the experimental study^9^ in which differential capacitance measurements allowed subtracting the parasitic capacitance. We then computed the dielectric constant at the level of water layers by integrating eqn (4) over single molecular layers at various distances from the surface. To this end, we divided the water slab into water layers parallel to the surface, as indicated by the dashed vertical lines in Fig. 1c (labeled L1, L2, L3, *etc.*), and computed *ε*_⊥_ for each of them. The first layer, L1, has a dividing surface centered at the carbon (boron) atom position (see ESI Fig. S4†) and is larger than the inner ones (∼4.7 Å *vs.* ∼3.3 Å) because it includes the depletion region between water molecules and graphene (hBN).

Fig. 1c shows the calculated interfacial water-layer *ε*_⊥_ at increasing distances from graphene using both AIMD and FFMD calculations. DFT (filled symbols) yields *ε*_⊥,L1_ ≈ 2.9 for the first layer, L1 (red colored area), which falls within the experimental error. For the second interfacial layer, L2 (blue colored area), it yields a slightly larger value, *ε*_⊥,L2_ ≈ 11, however remaining almost one order of magnitude smaller than the bulk value. We repeated our AIMD calculations of *ε*_⊥_ for water confined within hBN slits. Despite the different electronic structure of the confining material, we found no substantial differences in the computed values as compared to the graphene slits (not shown – see ESI Table S2†). This is to some extent expected, given that the structure of the water/graphene and water/hBN interfaces is extremely similar – see below and previous studies.^35,41^ Nonetheless, this is an important result, as it validates the assumption made in the experimental study, in which measurements were taken on asymmetric slits, that is, with graphite on one side of the slit and hBN on the other.^9^

We next turned to the force-field calculations (open symbols). The computed *ε*_⊥_ for the two interfacial layers is again in reasonable agreement with the experiments, yielding much reduced values relative to bulk with *ε*_⊥,L1_ ≈ 3.7 and *ε*_⊥,L2_ ≈ 16 for L1 and L2, respectively. These values are larger than the AIMD values approximately by a factor of 1.3 to 1.5, and depend little on the particular water–carbon parameterization used (see ESI Fig. S5†). Note that in the FFMD values shown in Fig. 1 we included the contribution of the electronic polarizability in order to compare them with the AIMD values. To do that, the high-frequency (electronic) dielectric constant of water, *ε*_∞_ ≈ 1.8, has been accounted for by adding 0.8 to the calculated values, as FFMD simulations with non-polarizable atoms yield high-frequency dielectric constant equal to 1, as pointed out in ref. 42. Importantly, our FFMD calculations show that water clearly recovers the bulk dielectric response beyond the first two interfacial layers. This reveals that water's dielectric response is insensitive to the presence of a surface at distances *z* > 7.5 Å, in agreement with the experimental findings. This is also consistent with recent theoretical calculations of another observable (the confined water function).^43^ Furthermore, the simulations for various thicknesses of the water slab (*h* = 1.935 [M], 3.0 [L] and 4.5 nm [XL]) show that the computed interfacial *ε*_⊥_ for L1 and L2 are essentially independent of the slit thickness as long as a bulk water phase exists in the system. This demonstrates that the observed polarization suppression does not arise from the confinement between the two surfaces, rather it is an intrinsic property of interfacial water molecules at each individual surface. We also ran simulations for various bulk water densities and verified that they have little effect on the predicted values (see ESI Fig. S6†).

Having calculated the dielectric constant of the interfacial water layers, we computed the effective dielectric constant over the whole water slab inside the slit as a function of the distance between the confining surfaces, *h*, as measured in the experiment, and directly compared it with the experimental results in Fig. 1d. To this end, we modeled the interface by three capacitors in series as in ref. 9.6
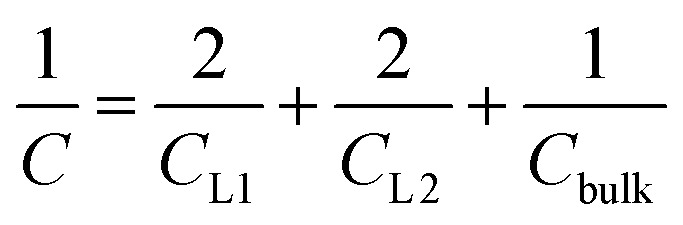
where *C* is the total capacitance, *C*_L1_ and *C*_L2_ the capacitance of the layers L1 and L2, respectively, and *C*_bulk_ the capacitance of the remaining bulk water (see Fig. 1a), where the factor 2 arises from having two interfaces in the slit. The dielectric constant of the water slab is thus calculated as7
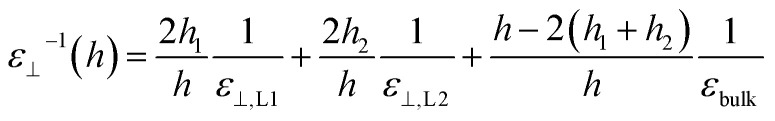
where *h*_1_ = 4.72 Å and *h*_2_ = 3.15 Å are the thickness of the interfacial layers L1 and L2, respectively, and *ε*_⊥,L1_ and *ε*_⊥,L2_ their dielectric constants previously calculated and shown in Fig. 1a. Fig. 1d displays the results of our calculations for both graphene (black) and hBN slits (cyan) using AIMD (filled symbols) and FFMD simulations (open symbols). We found that our AIMD prediction describes the experimental results with a fairly good accuracy. Water's dipolar polarization in ultrathin water slabs is strongly suppressed, with *ε*_⊥_ ≈ 2.9 for *h* ≃ 1 nm [S], which slightly increases up to *ε*_⊥_ ≈ 5 for *h* ≃ 2 nm [M]. For thicker water slabs, an intermediate regime is observed, with *ε*_⊥_ increasing linearly with *h* and recovering the bulk value only for 100 nm-thick water slabs, consistently with the experiment. Our force-field calculations predict a similar trend as a function of *h*, but yield slightly larger values, following from the offset found in the values of *ε*_⊥_ for the interfacial molecular layers (L1 and L2). We note that our FFMD calculations agree with recent reports in the literature^8,12,14,44^ (also see ESI Fig. S5†). Importantly, no significant differences were found between graphene and hBN slits, again as expected from the identical values of *ε*_⊥_ obtained for L1 and L2, as discussed above.

**Fig. 2:**
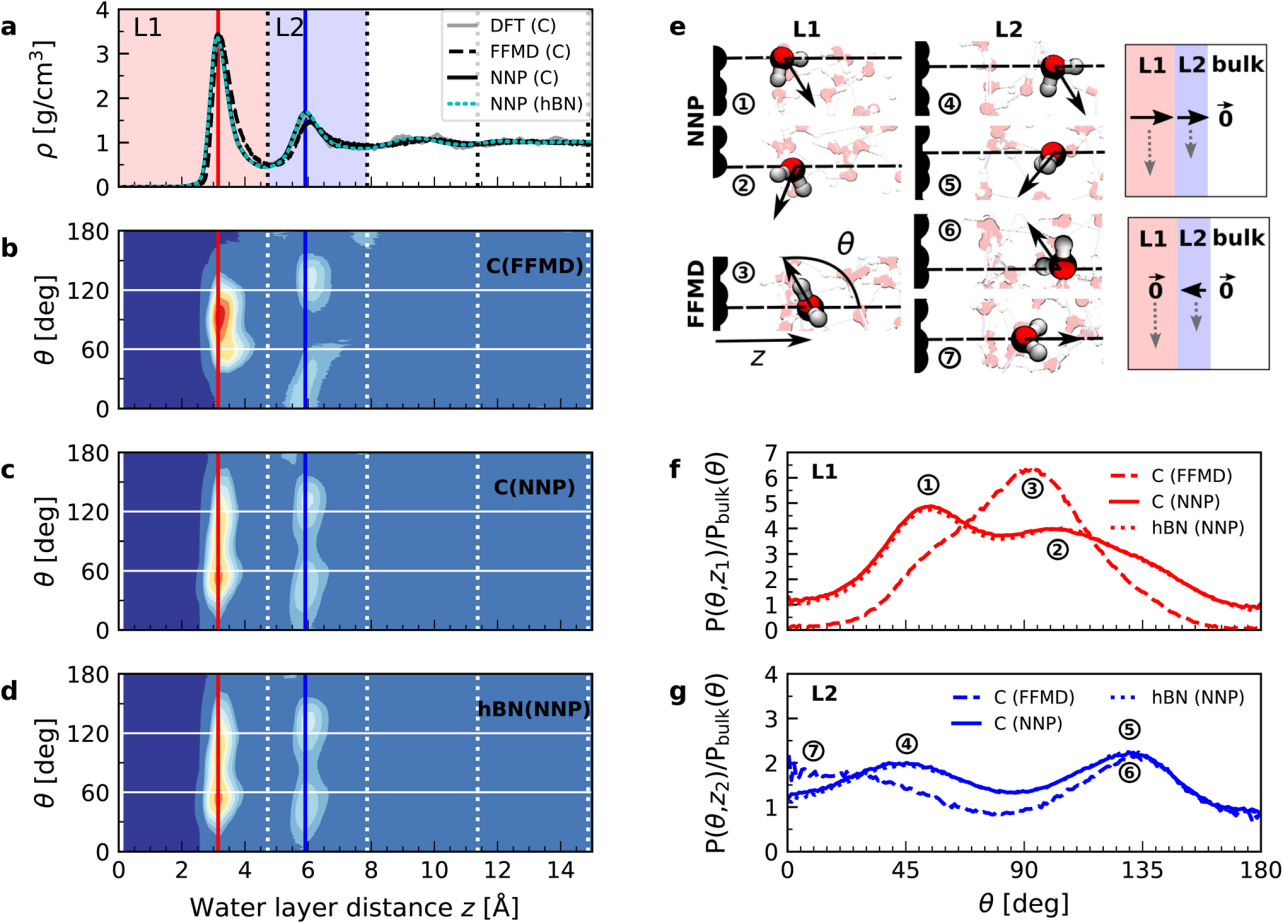
Orientation of water dipole moment near graphene and hBN surfaces. (a) Mass density profile obtained from AIMD (gray solid line), NNP (black solid line) and force-field (dashed black line) calculations for water on graphene and NNP calculations (cyan dashed line) for water on hBN for *h* = 3.0 nm [L]. AIMD profiles are obtained from the average of profiles computed from +*D* and −*D* simulations. (b) Distribution of the water dipole moment angle with respect to the perpendicular direction as a function of the distance from the surface for graphene using force-field calculations for *h* = 3.0 nm [L]. (c and d) Same as (b) but using NNP calculations for (c) graphene and (d) hBN. (e) Snapshots of water dipole orientation in the first and second water layers, L1 and L2, at graphene surfaces using NNP calculations (labels 1, 2, 4 and 5), showing antiparallel orientation in the perpendicular direction, and FFMD simulations (labels 3, 6 and 7). The FFMD snapshot corresponding to configuration (3) shows the definition of *z* and *θ*. The schematics on the right show the resulting net dipole moment in the normal direction (black solid arrows) in L1, L2 and the bulk for NNP (top) and FFMD (bottom) calculations (dashed gray arrows are the net dipole in the in-plane direction). (f and g) Dipole orientation distribution profiles at distance (f) *z* = 3 Å (L1) and (g) *z* = 6 Å (L2), corresponding to the red and blue vertical dashed lines in (b–d), for graphene (solid line) and hBN (dotted line) using NNP calculations. The dashed line indicates force-field calculations. The distributions are normalized by the isotropic distribution of bulk water with density of 1 g cm^−3^.

In order to understand the origin of the observed polarization behavior of the interfacial water molecules, we analyzed the structure and the electronic properties of the nanoconfined water slabs. To this end, we obtained converged structural insight from long-time simulations using neural network potentials (NNPs),^45,46^ which deliver DFT accuracy at zero field at substantially reduced computational cost. The NNPs for water confined by graphene and hBN were trained and validated following recent methodological developments^47^ and applied for extended simulations at various confinement widths (see Materials and methods). This enabled us to calculate in detail the time-averaged structural arrangement of water molecules and hydrogen-bonding network as a function of the distance from the confining surfaces. Fig. 2 shows some of these results at both graphene and hBN interfaces. As expected, we found no differences in the mass density profiles of water near graphene and hBN (Fig. 2a), which show two density maxima corresponding to the two interfacial layers (L1 and L2). Notably, the time-averaged orientational distributions obtained here reveal that water also has very similar orientations near graphene (Fig. 2c) and hBN (Fig. 2d). Indeed, there is an almost quantitative match in L1 and L2, as verified by plotting the distribution profile at the first two density maxima (Fig. 2f and g, respectively). We note that this is quite different for the in-plane alignment of the contact layer of water above these two materials, revealing distinct differences between graphene and hBN, as recently analysed in the context of water flow in nanotubes.^41^ Upon examining the orientation of the water molecules in the interfacial layers we find a broad distribution of orientations, falling broadly into two preferential directions in both L1 and L2. Specifically, taking *θ* as the angle between the surface normal and the water molecule dipole (see Fig. 2e), we find that in the first layer, the water dipole moments are oriented mainly either towards the bulk water at *θ* ≈ 60° or mainly towards the surface at *θ* ≈ 105°. These two preferential orientations are also found in the second layer, with a peak at *θ* ≈ 45° and the other at *θ* ≈ 135°. Thus, the molecules in the first two interfacial layers are loosely divided into two anti-parallel populations with respect to the dipole projection on the surface normal: either leaning towards the surface or towards the bulk.

We found that the effective dipolar polarization in the perpendicular direction that could be derived from the NNP simulations is greatly reduced compared to the water molecular dipole, in agreement with the much reduced dielectric response that was obtained here and in the experiment. This is consistent with previous simulations, indicating anticorrelated polarization of neighboring water molecules.^48^ It also confirms the recent suggestion of antiparallel perpendicular components of interfacial dipoles^49^ and the proposed Ising model, where however the perpendicular dipole moment can only take two values, positive and negative,^50^ at the origin of water's polarization suppression near the surface. The present study allows us to expand on this model by giving insights into which values the perpendicular moment can take in each water layer, and what constraint should be put on the total layer dipole in the absence of an external field. While an antiparallel arrangement of the interfacial dipoles is in agreement with our simulations, we observe broad distributions of dipoles with bimodular features. These distributions can be broken down into an antiparallel net orientation of the perpendicular components in the first two adsorption layers, not captured before. We also note that the force-field simulations tell a rather different story: consistent with previous force-field simulations,^12,22,25,51^ the dipoles in the first layer (Fig. 2b) are primarily oriented in a single dominant direction parallel to the surface. The second layer is more bimodal in nature but again different from the DFT distribution through the presence of molecules aligned precisely with the surface normal. These findings are robust with respect to the choice of the non-polarizable force field.

The dipole distribution profiles shown in Fig. 2f and g clearly show a breaking of symmetry in the two dipole orientations yielded by our NNP calculations. This translates into a residual net dipole moment in the perpendicular direction directed towards the bulk water in both the first and second interfacial layers, as sketched in Fig. 2e. This net polarization persists in the absence of an externally applied field, therefore it is ferroelectric in character and generates a local Maxwell field. We estimate the average field generated by the spontaneous polarization of the interfacial water to be around 6.5 V nm^−1^ in the first layer and 3.0 V nm^−1^ in the second layer using the relation ***E***(*z*) = −4π***P***(*z*) valid in the absence of an external electric displacement. We note that the force-field calculations also predict a ferroelectric order of interfacial water, but different from the one obtained by DFT calculations, also sketched in Fig. 2e. They yield a total net dipole only in the second layer oriented towards the surface, and not the bulk, while a net zero dipole is obtained in the first layer with the water dipoles oriented parallel to the surface.

**Fig. 3:**
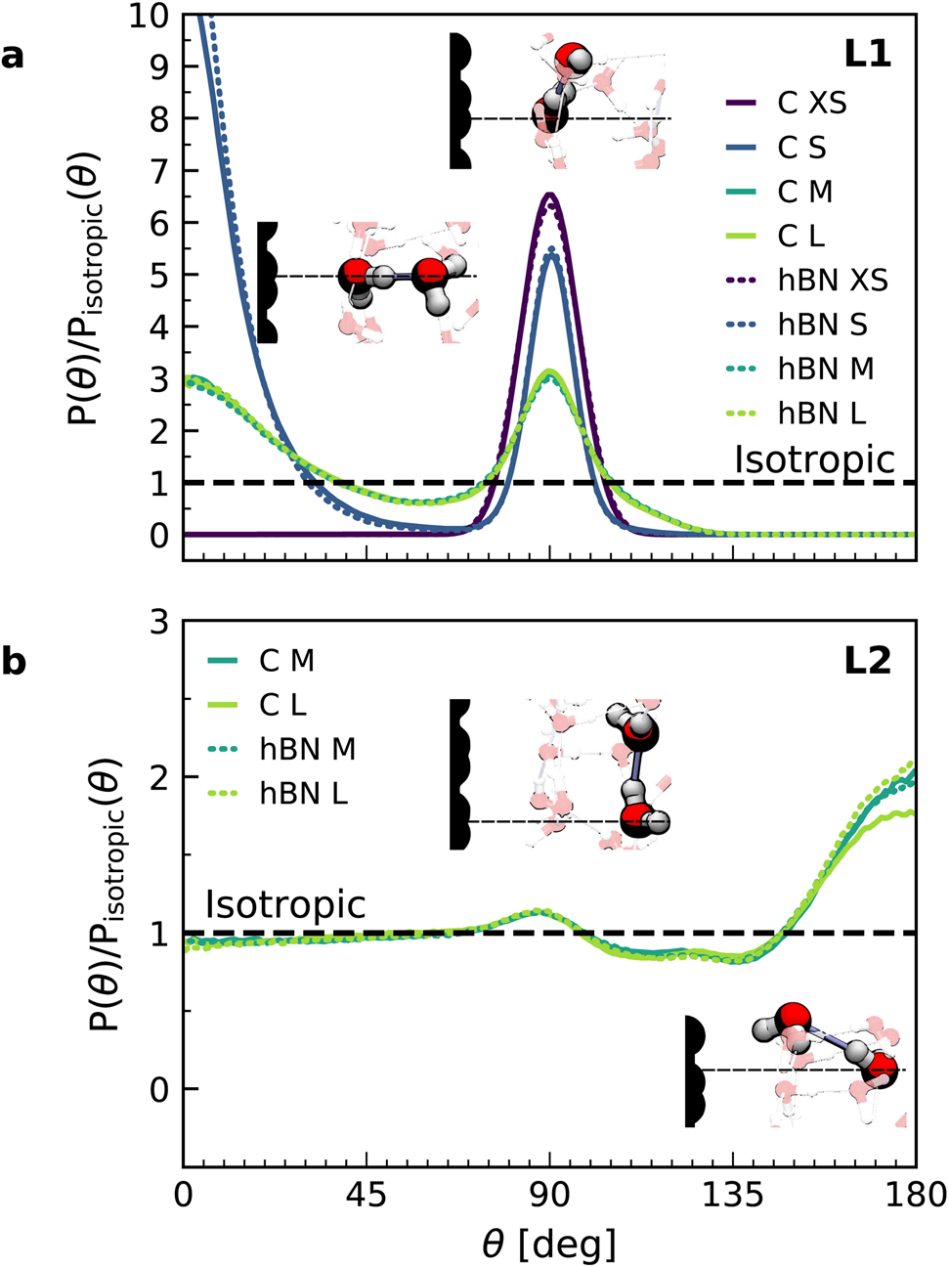
Orientation of hydrogen bonds in the interfacial water layers. Calculated orientation distribution with respect to the normal direction of the hydrogen bonds for water on graphene (solid lines) and hBN (dashed lines) in the first (a) and second (b) interfacial layers, normalized by the isotropic distribution, obtained from the combined NNP and DFT calculations (see FFMD simulations in ESI Fig. S6†). The direction corresponds to the donor oxygen–acceptor oxygen. Inset: snapshots of the main hydrogen bonding orientations.

Let us now analyze the nature of the hydrogen bonding network. Fig. 3 shows the orientation distribution of the hydrogen bonds, which we calculated from NNP simulations using a common hydrogen bond criterion^52^ (see details in ESI†). In the first layer, the hydrogen bond orientations are split into two populations: in-plane (*θ* ≈ 90°) and out-of-plane pointing towards the second layer (*θ* ≈ 0°). As expected, the latter disappears in the case of a water monolayer confined inside the slit (Fig. 3a, blue line), as the out-of-plane hydrogen bonds are absent. Similar orientations are also observed in force-field calculations (see ESI Fig. S7†). However, our NNP simulations yield a larger proportion of out-of-plane hydrogen bonds and in a smaller angular window, indicating a more constrained hydrogen-bonding network. The second layer displays an almost bulk-like behavior, with only a slight increase in in-plane hydrogen bonds, related to the dipole orientation at *θ* ≈ 130°, and in out-of-plane hydrogen bonds pointing towards the first layer.

**Fig. 4:**
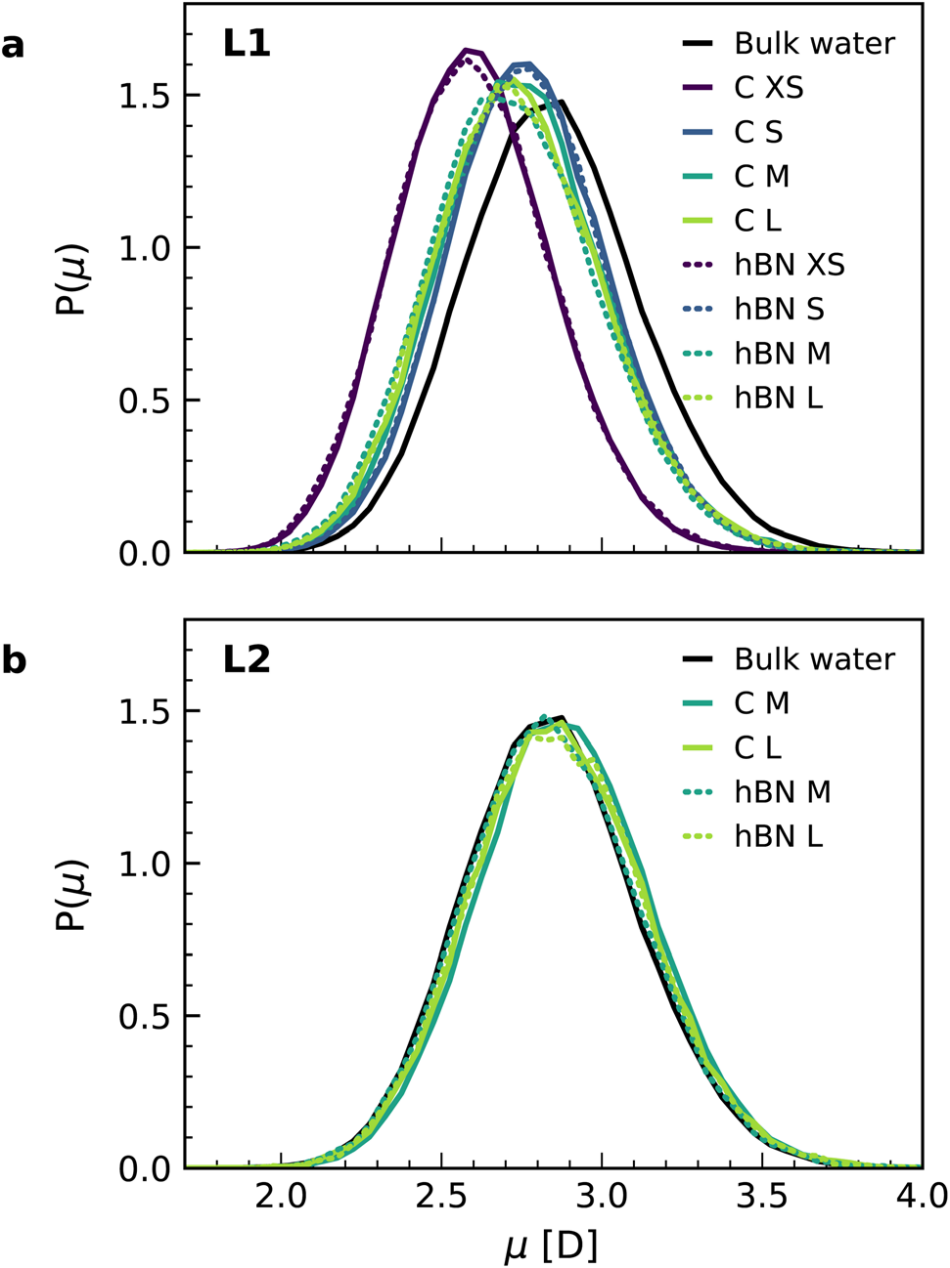
Dipole moment distribution in the interfacial water layers. Dipole moment distributions obtained from the combined NNP and DFT calculations for the first (a) and second (b) interfacial layers of water on graphene (solid lines) and hBN (dashed lines), as well as bulk water. The distribution is normalized by the isotropic distribution.

Finally, we calculated the molecular dipole moment in the first two interfacial layers using a combination of DFT and NNPs (Fig. 4) as it may also change with the change in the hydrogen-bonding network near the surface. Our results show a reduction in the dipole moment of the water molecules, but only in the first interfacial layer (Fig. 4a), not in the second one (Fig. 4b), with no appreciable dependence on the orientation of the water molecules. The decrease in the first layer (around 4%) is related to the decrease of the average number of hydrogen bonds per molecule in the first layer (2.93) as compared to the bulk (3.46), while this number remains bulk-like in the second layer (3.49). Notably, for the water monolayer confined inside the slit, it is more pronounced (around 9%) and is associated with a further decrease in the number of hydrogen bonds for each molecule down to 2.18. The observed decrease in the water molecular dipole contributes to the reduction of the dipolar polarization response and is not captured in the most commonly used force-field models, given the fixed nature of the water dipole moment. Although being a relatively minor contribution, this effect further accentuates the difference between first-principles and force-field interfacial water. As with the other properties, and despite the different electronic structures of the confining materials, we found no significant differences in the dipole moment distributions for water confined inside graphene and hBN slits.

## Discussion

In this study, we performed AIMD, AIMD-trained NNP simulations, and FFMD simulations of water confined within graphene and hBN slits aimed at understanding the out-of-plane dielectric constant of confined water. Our AIMD calculations reproduce the experimental results of ref. 9 with fairly good accuracy, yielding an interfacial layer of ∼7 Å thickness with reduced *ε*_⊥_ extending the first two interfacial water layers, an effect that is seen for both graphene and hBN interfaces and is independent of the distance between the confining surfaces of the slit. While in ref. 9 this effect was attributed to the preferential in-plane orientation of the interfacial water dipoles in the in-plane direction, in accordance with previously reported^51^ and more recent^12,22,25^ force-field simulations (also confirmed here), our AIMD and NNP MD simulations reveal a different origin. They indicate that it arises from two dominant effects: the spontaneous antiparallel alignment of the out-of-plane component of water dipoles, leading to a residual ferroelectric-like polarization of these two water layers, and the relatively constrained hydrogen bonds in the first two layers. Our simulations also reveal a small reduction in the dipole moments of the individual water molecules in the first layer. Taken together, these effects are responsible for the suppression of dipolar and hydrogen bond fluctuations in the out-of-plane direction, resulting in the anomalously low dielectric constant of confined water.

The value of *ε*_⊥_ obtained here from our DFT calculations quantitatively matches the experimental data within the experimental error for water layers extending 1–2 nm, that is, in the strong confinement regime. Notably, this is achieved by placing the dividing surface at the experimental position without the need of shifting it away from the water slab to match the experimental data, as discussed in the ESI.† However, for water slabs larger than 2 nm, in which bulk water molecules occupy the majority of the volume in the slits, we obtained values of *ε*_⊥_ that slightly overestimate the experimental data. This is reflected in the somewhat thicker “dead” layer reported in ref. 9, where the fitting of the experimental curve using the three-capacitor model yielded *ε*_⊥_ ≈ 2 for a layer extending ∼7 Å, that is, for both the first two interfacial layers, L1 and L2. In contrast, our simulations yield such a small *ε*_⊥_ only for L1, while for the second layer in contact with bulk water we obtained a larger value (*ε*_⊥_ ≈ 11) albeit remaining almost one order of magnitude smaller than that of bulk water. The latter confirms that the polarization suppression partially extends to the second interfacial layer far from the surface, and is not only limited to the first layer and the depletion region near the solid surface. The shift between this value and the experimental one is a relatively small difference, given the challenges of the experiment. However, it may also imply other effects not accounted for in our calculations are at play, as previously pointed out for force-field simulations.^14,20^ Two effects that have an impact on the quality of DFT predictions for water in general are the sensitivity of the results to the exchange–correlation functional and the importance of nuclear quantum effects (see *e.g.* ref. 53–58). To investigate these issues we examined our systems with a different exchange–correlation functional (PBE0-D4 as opposed to revPBED3) and with path integral molecular dynamics. As shown in the ESI† our key structural observations are not significantly changed by altering the functional or including nuclear quantum effects. Thus, the slight overestimate with respect to the experiment will require further theoretical and experimental work in the future to be fully understood.

The force-field simulations performed as part of this study also predict a reduced *ε*_⊥_ of confined water, in fairly good agreement with the experiments and previous studies.^8,12,14,44^ However, the extent of the reduction is underestimated compared to DFT. Partly this is down to the different interfacial water structures predicted by DFT and the force-fields, and partly because the force-field models fail to capture the small reduction in dipole moments of the interfacial (L1) water molecules. The purpose of the force-field simulations in the current study was primarily to compare these previously used methods to our DFT simulations, and so only simple point charge models were considered. Standard point charge force-field models, while being incredibly useful in deepening understanding of water in general, have been designed for bulk water and cannot be expected to deliver quantitative accuracy for interfacial water.

Finally, we note that the emergence of ferroelectricity in interfacial and confined water due to the spontaneous alignment of water dipoles near surfaces has been under debate for many years. In particular, water has been predicted to arrange in ferroelectric or antiferroelectric configurations when confined in one-dimensional channels such as nanotubes and nanopores^59,60^ and in 2D nanoslits in the in-plane direction,^61^ while experimental evidences remain scarce and are generally associated to ice phases.^62–64^ Our first-principles calculations provide insight into this question as they indicate that while retaining liquid-like mobility, interfacial water near hydrophobic surfaces is ferroelectric in the out-of-plane direction at room temperature, irrespective of the presence of two confining surfaces. Importantly, the electrically “dead” water layers reported in ref. 9 should arise from this effect. Note, however, that when water is under strong confinement between two hydrophobic surfaces, as in the experiment, the interfacial water at the two confining surfaces exhibits a net polarization towards the bulk water in the center of the slit (see sketch in Fig. 2e), thus ordering in opposite directions with respect to the surface normal in an overall antiferroelectric configuration in the slit. From the calculated net polarization, we estimate the coercive external field required to reorient it to be at least a few V nm^−1^, which is much larger than the electric field effectively applied to the water in the experiment, estimated to be around 0.05 V nm^−1^.^9^ This would explain why the interfacial water dipoles remained aligned in their spontaneous configuration near the surfaces in the experiment and did not reorient in the opposite direction with the external field. Given the general nature of confinement in our study and that much of surface chemistry and molecular biology is influenced by the dielectric properties of water at interfaces, we expect these findings to be of broader relevance, in particular for understanding water's polarization near and across biological membranes and for developing new energy storage/conversion and molecular sensing devices.

## Materials and methods

### *Ab initio* molecular dynamics calculations

AIMD simulations were performed with the CP2K^65^ code. We simulated boxes with a lateral size of 1.23 × 1.28 nm corresponding to 5 × 3 supercells of the orthorhombic unit cells of the two-dimensional confinement materials. These comprise 60 carbon atoms or 30 BN pairs per layer of graphene and hBN, respectively. We used the same lateral dimensions of the cell for both graphene and hBN due to their very similar lattice constants^66^ (see details in ESI Table S1†). The separation between the two confining surfaces, *h*, varied from 0.663 [XS], to 0.911 [S], to 1.935 nm [M]. A separation of 3.00 nm [L] has also been tried, but considering the slow convergence of the dielectric constant and the expensive computational cost associated with a larger number of water molecules, it was instead used to validate the NNP calculations. 2.0 nm of vacuum was introduced to prevent interactions between periodic images in the normal direction. We used the revPBE functional^67^ with the D3 (ref. 68) correction for dispersion interactions, given its good performance in accurately reproducing both the experimentally measured structure and dynamics of liquid water^69–71^ as well as the interaction energies of water on graphene and inside carbon nanotubes (CNTs) obtained using more advanced methods such as quantum Monte Carlo and coupled cluster theory.^28^ Despite the known weaknesses for the prediction of denser phases of ice,^72^ this particular choice of DFT functional provides an ideal compromise between efficiency and accuracy for the present work. Furthermore, we show in the ESI† that another reasonable choice for the DFT functional, in particular the hybrid functional PBE0 with D4 corrections, provides very similar results for the key interfacial properties of water in contact with both graphene and hBN. Goedecker–Teter–Hutter (GTH) pseudopotentials^73^ were used along with a DZVP basis set for carbon, boron, and nitrogen and a TZV2P basis set for oxygen and hydrogen in conjunction with a 750 Ry plane-wave cutoff. Simulations were performed in the NVT ensemble with a target temperature of 330 K using a Langevin thermostat, a timestep of 1.0 fs, and deuterium masses for the hydrogen atoms. The surface atoms were kept fixed and initial configurations were generated using the fftool and Packmol packages.^74^ These simulation cells were first equilibrated using FFMD calculations using the force field and simulation parameters described below. The number of water molecules was adjusted such that the mass density profiles match the ones obtained for the larger simulation cells used for the FFMD simulations described below. Each simulation was first equilibrated for 3 ps, while all shown properties were accumulated over at least 60 ps additional simulation time (see details in the ESI Fig. S3†). The dipole moment distribution of water was calculated with DFT using the same functional and set-up as described above. For this purpose we computed the position of the Wannier centers of the water molecules using uncorrelated structures obtained from the NNP trajectory (see below). Values of the dipole moments reported in Fig. 4 were obtained by averaging data obtained every 0.8 ps from the first 5 ns (0.66 nm [XS] and 0.91 nm [S]), 3 ns (1.94 nm [M]) and 2 ns (3.00 nm [L]) NNP trajectories described below.

### Force-field molecular dynamics calculations

We simulated boxes of lateral size of 3.76 nm with two confining layers at distance *h* 0.663 [XS], 0.911 [S], 1.935 [M], 3.00 [L] and 4.50 nm [XL] using the same lattice constant as in the AIMD simulations. Simulations were performed in the NVT ensemble at 300 K using a Nosé–Hoover thermostat with a time constant of 1 ps and a timestep of 2 fs. Each simulation was equilibrated for 2 ns, while data were accumulated for the following 5 ns. For simulations without an applied field, we used the LAMMPS package,^75^ with initial configurations generated using the fftool and Packmol packages.^74^ The number of water molecules was adjusted such that the density was 1 g cm^−3^. The density was defined as the mass of water molecules divided by the accessible volume, where the accessible volume corresponds to the region in space where the atomic number density (either oxygen or hydrogen) is non zero. For the simulations in the constant-*D* ensemble, we used the Metalwalls software.^76^ The water molecules were modeled with the SPCE model^77^ and three different water–carbon interaction potentials.^13,78,79^ All data shown in the main text were obtained with the water–carbon parameterization from.^13^ Data using other water–carbon models are provided in the ESI.† For the water–hBN interaction, we used the force-field of.^80^

### Dielectric constant calculations

The perpendicular dielectric constant was computed following the formalism of ref. 5 and using the finite-field method, applying an electric displacement of ±1.0 V nm^−1^ for DFT calculations and ±1.5 V nm^−1^ for FFMD calculations. The lower electric displacement value used in the DFT calculations was chosen to be safely within the linear regime, while this is estimated to be 1.5–2.0 V nm^−1^ for force-field calculations, showed by the emergence of non linearities at 2 V nm^−1^.^81^ Being conservative, we chose a slightly lower value for our DFT calculations. To improve the convergence, we computed the difference in polarization between positive and negative dielectric displacement, +*D* and −*D*, effectively doubling the window of the linear regime as explained in the main text. The dielectric displacement is applied only in the direction perpendicular to the confining surfaces using the same procedure as in ref. 33, implying 3D periodic boundary conditions, along with Ewald summation for the Coulomb interactions. It is based on the used of the extended Hamiltonian *H*_D_ = *H*_0_ + *V*/(8π)[*D* − 4π*P*]^2^, where *H*_0_ is the Hamiltonian in the absence of external field, and *V* is the volume of the simulation cell. In practice, it translates into an additional force on all charged particle *F*_D_ = *q*[*D* − 4π*P*] where *q* is the charge of the particle. In addition, for AIMD simulations the additional term in the Hamiltonian will affect the electron distribution. In cases of the force and electron distribution, the total polarization is computed from the Berry phase. For the AIMD calculations, we computed the local polarization from the full charge density (electrons plus nuclei), thus taking into account the multipole orders in the polarization derivation. To remove the contribution of the confining surfaces, we carried out a differential calculation, in which we subtracted the response of the confining surfaces without water to the local response of the total system (see details in the ESI Fig. S2†). As the atoms of the surface were kept fixed, only a single calculation was required to compute the former. In the case of AIMD calculations, the convergence of the dielectric profile proved to be slow (not converging after 70 ps of simulations). On the other hand, the dielectric constant converged when integrating the dielectric profile over a molecular layer, as discussed in details in the ESI (Fig. S3).† We note that the scale of the molecular layer (a slice with a width of around 0.4 nm and an infinite extension along the interface directions) is more relevant for this study, as eqn (1)–(4) have been derived in the scope of continuous media theory. Thus, we extracted the dielectric response of the first two interfacial layers, which tend to converge faster due to their small value, and the constant-*D* ensemble, which allowed us to directly obtain the inverse of the dielectric constant. Using the equations in the main text along with Maxwell–Gauss equation, it can be shown that working in the finite ***D*** ensemble, the averaged applied perpendicular field is equal to its local value, irrespective of the size of the simulation box. To limit the computational cost of AIMD calculations, we assumed bulk-like dielectric response for the remaining water molecules in the center of the slit, as found in the force-field calculations.

### Machine learning potentials

In order to access converged structural properties of water confined by graphene and hBN layers, we made use of machine learning potentials to perform long-time simulations at DFT accuracy. For that purpose, we utilized Behler–Parrinello neural network potentials (NNPs)^45,46^ in a committee model^82^ enabling the simple development^47^ of machine learning potentials at first-principle accuracy for the two confining materials. Using this methodology, we trained and validated the two models as described in detail in ref. 41 and applied them here for MD simulations at various surface separations, always comparing graphene and hBN confinement. As before, simulations were performed with CP2K in the NVT ensemble at 330 K using a Langevin thermostat, a timestep of 1.0 fs, deuterium masses for the hydrogen atoms and keeping the atoms of the confinement material fixed. Systems with two confining layers at distance *h* = 0.663 [XS], 0.911 [S], 1.935 [M], 3.00 [L] and a lateral size of 1.23 × 1.28 nm were propagated for 5 ns to converge structural properties. Furthermore, the NNPs allowed us to analyse the influence of the quantum nature of the nuclei with thermostatted ring polymer molecular dynamics simulations.^83^ These simulations provide access to rigorous quantum thermodynamical properties. We used a timestep of 0.25 fs and 16 ring polymer replica resulting in a converged description of structural properties.^84^ Simulations were performed for the 3.00 nm [L] system over a simulation length of 500 ps for both confining materials. Detailed comparison of all analysed properties of interfacial water with classical and quantum nuclei is provided in the ESI.† These results show that nuclear quantum effects have negligible impact on the key findings of this study. Two additional models trained to PBE0-D4 were used to assess the impact of the DFT functional on the interfacial properties of water in contact with both graphene and hBN. Similar to the impact of nuclear quantum effects, the choice of DFT functional does not significantly impact the properties of interfacial water, as discussed in detail in the ESI.†

## Data availability

The ESI† provides additional computational details. Simulation data will also be made available on the Cambridge Apollo repository prior to publication.^85^

## Author contributions

L. F., T. D., C. S. and A. M. designed the research; T. D. performed the simulations and C. S. developed the machine learning models; all authors analyzed the data; and L. F., T. D., C. S., and A. M. wrote the paper, with contributions from J. C. and A. K. G.

## Conflicts of interest

The authors declare no competing financial or non-financial interests.

## Supplementary Material

SC-015-D3SC04740G-s001
