# Supplementary material for: Origin of dielectric polarization suppression in confined water from first principles
Source: Chem Sci. 2023 Dec 4;15(2):516–27. doi: 10.1039/d3sc04740g (PMC10763014; doi:10.1039/d3sc04740g)
Supplement: SC-015-D3SC04740G-s001 [file SC-015-D3SC04740G-s001.pdf]

Electronic Supplementary Material (ESI) for Chemical Science.
This journal is © The Royal Society of Chemistry 2023

Supplementary Information

Contents

S1 Simulation details **S2**

S2 Dielectric constant calculations **S3**

S2.A Polarization computation . . . S3
S2.B Subtracting the surface contribution . . . S3
S2.C Data convergence . . . S4
S2.D Impact of the dividing surface . . . S5

S3 Additional force-field simulations **S7**

S4 Hydrogen bond orientation **S9**

S5 Influence of DFT Functional **S11**

S6 Influence of Nuclear Quantum Effects **S12**

S1 Simulation details

All relevant information about the simulations performed in this study are given in Table S1 below.

Table S1: Details of the AIMD FFMD and NNP simulations. For each slit thickness h, defined as the separation between the two confining materials, we provide the cell dimensions $L_{x/y/z}$, the number of wall atoms N_{wall} and water molecules N_{water}, the equilibration period t_{eq} and the total simulation length t_{sim}.

Slit thickness	Simulation details		
	Graphene (AIMD/NNP)	hBN (AIMD/NNP)	Graphene (FFMD)
XS<br>$h = 6.63\,\text{Å}$	$L_x = 12.30\,\text{Å}$<br>$L_y = 12.78\,\text{Å}$<br>$L_z = 26.63\,\text{Å}$<br>$N_{\text{wall}} = 120$<br>$N_{\text{water}} = 16$<br>$t_{\text{eq}}^{\text{DFT}} = 3\,\text{ps}$<br>$t_{\text{sim}}^{\text{DFT}} = 100\,\text{ps}$<br>$t_{\text{eq}}^{\text{NNP}} = 250\,\text{ps}$<br>$t_{\text{sim}}^{\text{NNP}} = 5\,\text{ns}$	$L_x = 12.30\,\text{Å}$<br>$L_y = 12.78\,\text{Å}$<br>$L_z = 26.63\,\text{Å}$<br>$N_{\text{wall}} = 120$<br>$N_{\text{water}} = 16$<br>$t_{\text{eq}}^{\text{DFT}} = 2\,\text{ps}$<br>$t_{\text{sim}}^{\text{DFT}} = 100\,\text{ps}$<br>$t_{\text{eq}}^{\text{NNP}} = 250\,\text{ps}$<br>$t_{\text{sim}}^{\text{NNP}} = 5\,\text{ns}$	$L_x = 36.89\,\text{Å}$<br>$L_y = 38.34\,\text{Å}$<br>$L_z = 80.0\,\text{Å}$<br>$N_{\text{wall}} = 1080$<br>$N_{\text{water}} = 147$<br>$t_{\text{eq}} = 2\,\text{ns}$<br>$t_{\text{sim}} = 5\,\text{ns}$
S<br>$h = 9.11\,\text{Å}$	$L_x = 12.30\,\text{Å}$<br>$L_y = 12.78\,\text{Å}$<br>$L_z = 29.11\,\text{Å}$<br>$N_{\text{wall}} = 120$<br>$N_{\text{water}} = 30$<br>$t_{\text{eq}}^{\text{DFT}} = 2.5\,\text{ps}$<br>$t_{\text{sim}}^{\text{DFT}} = 80\,\text{ps}$<br>$t_{\text{eq}}^{\text{NNP}} = 250\,\text{ps}$<br>$t_{\text{sim}}^{\text{NNP}} = 5\,\text{ns}$	$L_x = 12.30\,\text{Å}$<br>$L_y = 12.78\,\text{Å}$<br>$L_z = 29.11\,\text{Å}$<br>$N_{\text{wall}} = 120$<br>$N_{\text{water}} = 30$<br>$t_{\text{eq}}^{\text{DFT}} = 2\,\text{ps}$<br>$t_{\text{sim}}^{\text{DFT}} = 80\,\text{ps}$<br>$t_{\text{eq}}^{\text{NNP}} = 250\,\text{ps}$<br>$t_{\text{sim}}^{\text{NNP}} = 5\,\text{ns}$	$L_x = 36.89\,\text{Å}$<br>$L_y = 38.34\,\text{Å}$<br>$L_z = 80.0\,\text{Å}$<br>$N_{\text{wall}} = 1080$<br>$N_{\text{water}} = 268$<br>$t_{\text{eq}} = 2\,\text{ns}$<br>$t_{\text{sim}} = 5\,\text{ns}$
M<br>$h = 19.35\,\text{Å}$	$L_x = 12.30\,\text{Å}$<br>$L_y = 12.78\,\text{Å}$<br>$L_z = 39.35\,\text{Å}$<br>$N_{\text{wall}} = 120$<br>$N_{\text{water}} = 82$<br>$t_{\text{eq}}^{\text{DFT}} = 4\,\text{ps}$<br>$t_{\text{sim}}^{\text{DFT}} = 120\,\text{ps}$<br>$t_{\text{eq}}^{\text{NNP}} = 250\,\text{ps}$<br>$t_{\text{sim}}^{\text{NNP}} = 5\,\text{ns}$	$L_x = 12.30\,\text{Å}$<br>$L_y = 12.78\,\text{Å}$<br>$L_z = 39.35\,\text{Å}$<br>$N_{\text{wall}} = 120$<br>$N_{\text{water}} = 82$<br>$t_{\text{eq}}^{\text{DFT}} = 2\,\text{ps}$<br>$t_{\text{sim}}^{\text{DFT}} = 120\,\text{ps}$<br>$t_{\text{eq}}^{\text{NNP}} = 250\,\text{ps}$<br>$t_{\text{sim}}^{\text{NNP}} = 5\,\text{ns}$	$L_x = 36.89\,\text{Å}$<br>$L_y = 38.34\,\text{Å}$<br>$L_z = 80.0\,\text{Å}$<br>$N_{\text{wall}} = 1080$<br>$N_{\text{water}} = 754$<br>$t_{\text{eq}} = 2\,\text{ns}$<br>$t_{\text{sim}} = 5\,\text{ns}$
L<br>$h = 30.0\,\text{Å}$	$L_x = 12.30\,\text{Å}$<br>$L_y = 12.78\,\text{Å}$<br>$L_z = 30.00\,\text{Å}$<br>$N_{\text{wall}} = 120$<br>$N_{\text{water}} = 140$<br>$t_{\text{eq}}^{\text{DFT}} = 2\,\text{ps}$<br>$t_{\text{sim}}^{\text{DFT}} = 24\,\text{ps}$<br>$t_{\text{eq}}^{\text{NNP}} = 250\,\text{ps}$<br>$t_{\text{sim}}^{\text{NNP}} = 5\,\text{ns}$	$L_x = 12.30\,\text{Å}$<br>$L_y = 12.78\,\text{Å}$<br>$L_z = 30.00\,\text{Å}$<br>$N_{\text{wall}} = 120$<br>$N_{\text{water}} = 140$<br>$t_{\text{eq}}^{\text{NNP}} = 250\,\text{ps}$<br>$t_{\text{sim}}^{\text{NNP}} = 5\,\text{ns}$	$L_x = 36.89\,\text{Å}$<br>$L_y = 38.34\,\text{Å}$<br>$L_z = 90.65\,\text{Å}$<br>$N_{\text{wall}} = 1080$<br>$N_{\text{water}} = 1258$<br>$t_{\text{eq}} = 2\,\text{ns}$<br>$t_{\text{sim}} = 5\,\text{ns}$
XL<br>$h = 45.0\,\text{Å}$			$L_x = 36.89\,\text{Å}$<br>$L_y = 38.34\,\text{Å}$<br>$L_z = 105.65\,\text{Å}$<br>$N_{\text{wall}} = 1080$<br>$N_{\text{water}} = 1968$<br>$t_{\text{eq}} = 2\,\text{ns}$<br>$t_{\text{sim}} = 5\,\text{ns}$

S2 Dielectric constant calculations

S2.A Polarization computation

For all AIMD estimates of the dielectric constant reported in the main text, we computed the polarization from the charge density of our AIMD simulations. This assumes that the polarization for $z = 0$ is equal to 0, given that all atoms are far away. To validate this approach, we computed the total dipole moment of the simulation box for various configurations over a 500 fs simulation at an applied electric displacement of 1 Vnm^{-1} and $h = 1.935$ nm [S]. The dipole moments were computed every 20 fs. Figure S1 contrasts the values obtained from the charge density as described in the main text, the Berry phase and the Wannier centers. It shows that $P(z = 0) = 0$ is a very good assumption and allows the computational cost of the Wannier centers to be avoided. The error made is of the order of 3.10^{-3} atomic units for the dipole moment. Deriving the error on the polarization and using Eq.(4) of the main text, this leads to an error on the inverse of the dielectric constant on the order of 10^{-3}, thus validating our choice for computing the polarization.

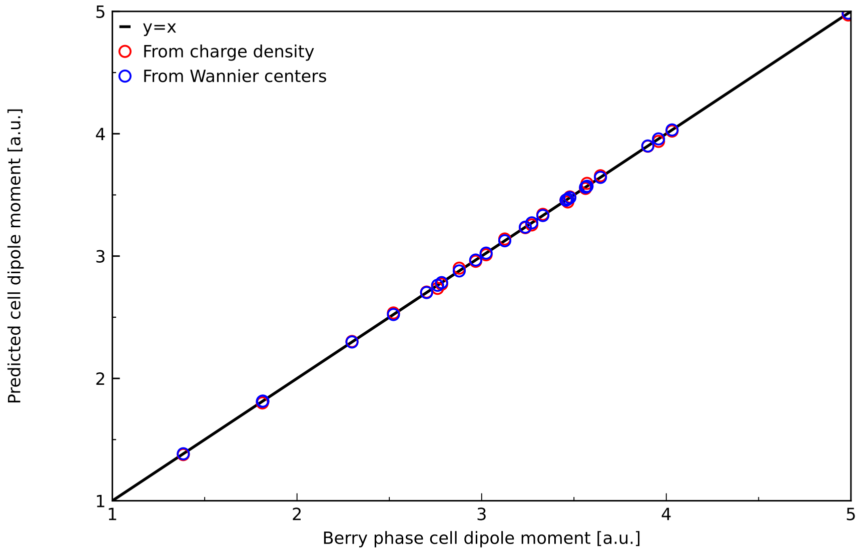

Figure S1: Computation of the dipole moment. Correlation between the dipole moment of the simulation cell computed from the Berry phase, the Wannier centers and the charge density.

S2.B Subtracting the surface contribution

In the AIMD simulations, the confining surfaces of the slits (graphene and hBN) contribute to the global polarization response due to the extension of their electronic clouds that partially overlaps with the one of water. In experiments, the confining material contributes also to the dielectric response, and its contribution is removed by performing a differential measurement [1]. Thus, we used a similar approach here, where we corrected the calculated values by subtracting the local response of the system in the absence of water, that is composed only of the confining surfaces (see snapshots in Fig. S1a-c). First, we extracted the total local polarization response ΔP_z^{tot} from the total system. We then extracted the polarization response of the confining surfaces ΔP_z^{CS} from a single calculation without water. This enabled us to obtain an approximation of the water response as $\Delta P_z^{H2O} = \Delta P_z^{tot} - \Delta P_z^{CS}$. By combining it with the expression of the local dielectric constant, we get

$$\left[\epsilon_\perp^{H2O}(z)\right]^{-1} = \left[\epsilon_\perp^{tot}(z)\right]^{-1} + \left(1 - \left[\epsilon_\perp^{CS}(z)\right]^{-1}\right). \quad (1)$$

The result of this procedure is reported in Fig. S2 below, showing the dielectric constant profile obtained calculating a single configuration for the case of graphene slits (Fig. S2, profiles on the left) and hBN slits (Fig. S2, profiles on the right). It shows that the contribution of the confining material to the dielectric response has been replaced by the one of vacuum.

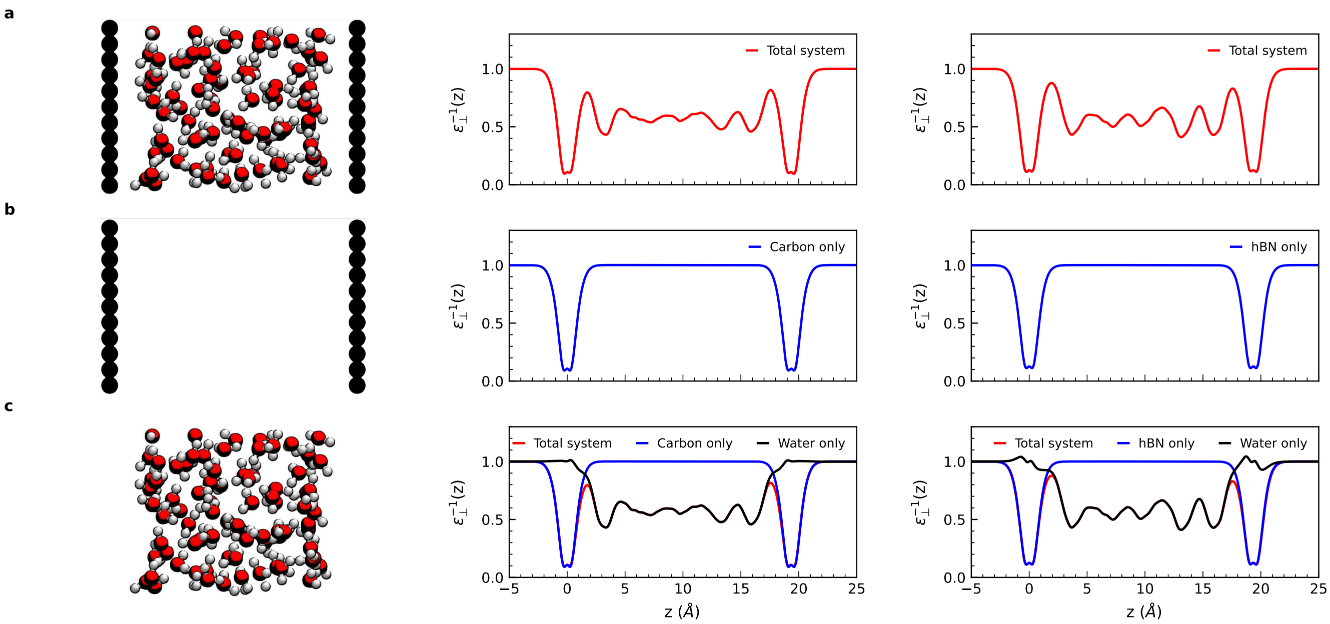

Figure S2: Calculation of water's dielectric constant profiles. Snapshots (left) and corresponding dielectric profiles (right) for the total system (a), the system without water (b) and the profile (c) obtained after subtracting the polarization response of the graphene layer (left) and hBN layer (right) to the total system.

S2.C Data convergence

To check the equilibration of the simulations, we analyzed the time evolution of the dipole moment of the simulation boxes along with its running average, shown in Fig. S3a below for water inside a graphene slit for the M system. The convergence of the total dipole moment was found to be very fast, as reported in a previous study on bulk water [2]: equilibration and convergence is faster at finite D than at finite E.

We calculated the dielectric constant of the water layers reported in Fig. 1b in the main text from the running average of the dielectric constant, shown in Fig. S3b below, by taking the average of $\epsilon_\perp^{-1}$ in the long-time limit, corresponding to the last 20 ps of each simulation, except for the S [0.911 nm] system where the calculations were made on the last 10 ps. For the L [3.00 nm] system, we used the last 60 ps, as the response of the second layer requires more statistics to converge. Even if Fig.S3b shows that the dielectric constant is not fully converged for the 1.935 nm [M] slit size, we have used it to compute the dielectric constant as it is at the limit of what can currently be reached with AIMD simulations. The error was estimated from the fluctuations of the running value of $\epsilon_\perp^{-1}$ around this average, and propagated to obtain the error in $\epsilon_\perp$.

We summarized the obtained dielectric constants of the first and second water layers (L1 and L2, respectively) for graphene and hBN slits in Table S2 below. For graphene, we also computed the dielectric constant $\epsilon_\perp^{C,PCD}$ by assuming that the charge distribution corresponds to the one of the SPCE model, i.e. a point charge distribution

Table S2: Dielectric constant of interfacial water layers. Calculated dielectric constants of the interfacial water layers, L1 and L2, reported in Fig. 1b in the main text, for both graphene (first column) and hBN slits (second column) and various water slab thickness using AIMD simulations. The third column computes the dielectric constant from the AIMD configurations by assuming that the charge distribution corresponds to the one of the SPCE model, i.e. a point charge distribution. The error is obtained from the fluctuations of the running average in the long-time limit.

h (nm)	$\epsilon_\perp^{C}$	$\epsilon_\perp^{hBN}$	$\epsilon_\perp^{C,PCD}$
	L1		
1.935	2.94 ± 0.12	2.80 ± 0.12	2.80 ± 0.33
0.911	2.874 ± 0.013	2.871 ± 0.015	3.38 ± 0.16
0.663	1.7805 ± 0.0054	2.344 ± 0.010	2.116 ± 0.039
	L2		
1.935	10.55 ± 1.62	11.07 ± 8.03	7.80 ± 2.76

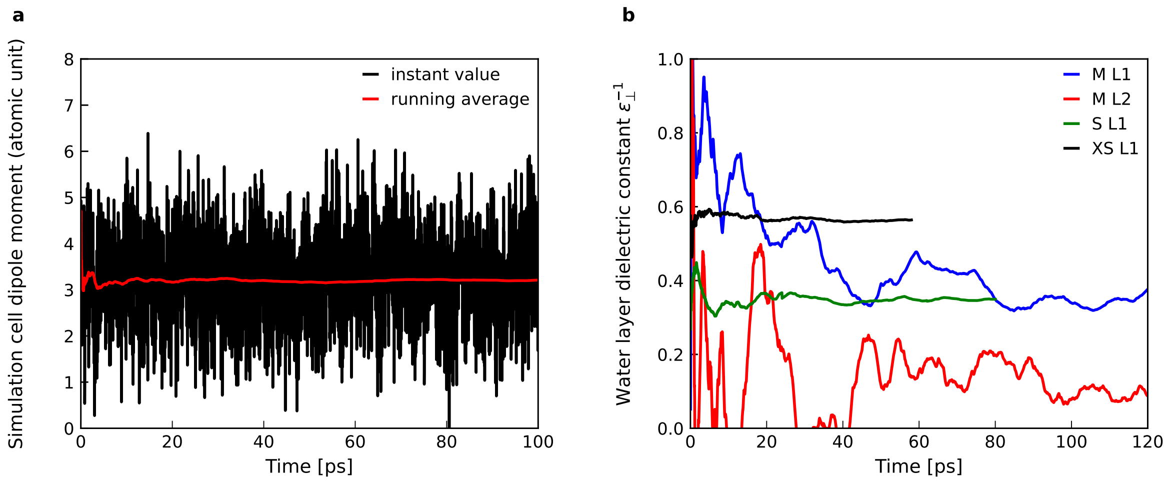

Figure S3: Convergence test of water's dielectric properties in AIMD simulations. Convergence of the cell dipole moment calculated using the Berry phase for an electric displacement of -1.0 V.nm^{-1} (a) with the corresponding running average of the total dipole moment for the M [1.935 nm] system and (b) running value of the dielectric constant of the interfacial water layers L1 and L2 for different water slab thicknesses.

(PCD), in order to estimate the relative proportion of orientation and electronic effects. We found that the obtained values of $\epsilon_\perp$ for graphene and hBN slits were equal within the error bar, except for the monolayer configuration which exhibits a slightly lower value for graphene. We obtained larger uncertainties in the dielectric constant for the second layer, L2, which we attribute to two factors. First, it arises from the higher error sensitivity of these calculations for higher values of $\epsilon_\perp$, that is, for a given uncertainty in $\epsilon_\perp^{-1}$, the uncertainty in $\epsilon_\perp$ increases with increasing $\epsilon_\perp$. Secondly, this may arise from the more dynamical character of the system with larger slit thickness, with more molecules exchanging between the layers that slow down the convergence. The comparison with the dielectric constant obtained from the point charge distributions shows that most of the difference between the FFMD and AIMD calculations comes from the difference in the molecular orientation rather than electronic effects.

To understand the impact of the choice of DFT functional, we repeated the DFT simulations for the 1.935 nm [M] graphene slit using the BLYP functionnal with the D3 correction. These calculations yielded a dielectric constant for the first interfacial water layer (L1) of 2.096 ± 0.067, a value in agreement with and even lower than what we found in this study. We note however that the response of the L2 layer did not converge even with 120 ps simulations.

S2.D Impact of the dividing surface

The dividing surface, that is, the domain boundary over which the dielectric profile is integrated, is not well defined on the atomic scale due to the non-continuous nature of the interface between the confining layer and the water phase. We investigated the effect of the choice of the dividing surface by considering four positions, indicated in Fig. S4a (dashed lines): the confining surface (CS), the one used in the main text corresponding to the atomic position of the confining layer (e.g. carbon atoms for graphene); the electronic density crossover (EC), corresponding to the local density maximum between the carbon wall and the water phase; the full density (FD) surface, corresponding to the symmetric position of the EC surface with respect to the confining surface; and the jellium edge (JE) [3], corresponding to the extension of the electronic cloud of the confining material in a mean-field approach.

Figure S4b shows the calculated inverse dielectric constant for each position of the dividing surface against the experimental data as a function of the water slab thickness. The calculated values are sensitive to the dividing surface position, and this is due to the change in the domain volume, as the dipole moment remains largely unchanged. In particular, shifting the dividing surface from the atomic position towards the vacuum, as in the case of the FD surface, increases the volume at constant dipole moment, thus decreasing the resulting polarization, which in turn leads to a decrease in the obtained $\epsilon_\perp$. The opposite occurs when the dividing surface is shifted towards the liquid phase, as in the case of EC and JE surfaces: the polarization is increased with decreasing the volume, and in turn $\epsilon_\perp$ increases up to infinity and even becomes negative. In this study we show that our calculations yield values of $\epsilon_\perp$ in reasonably good agreement with the experimental values by simply taking the dividing surface at its physical position - the atomic position of the confining layer (CS) - and subtracting the electronic cloud contribution beyond the surface, as explained in section S2.A above, without any need of shifting the dividing surface to a different position. We note

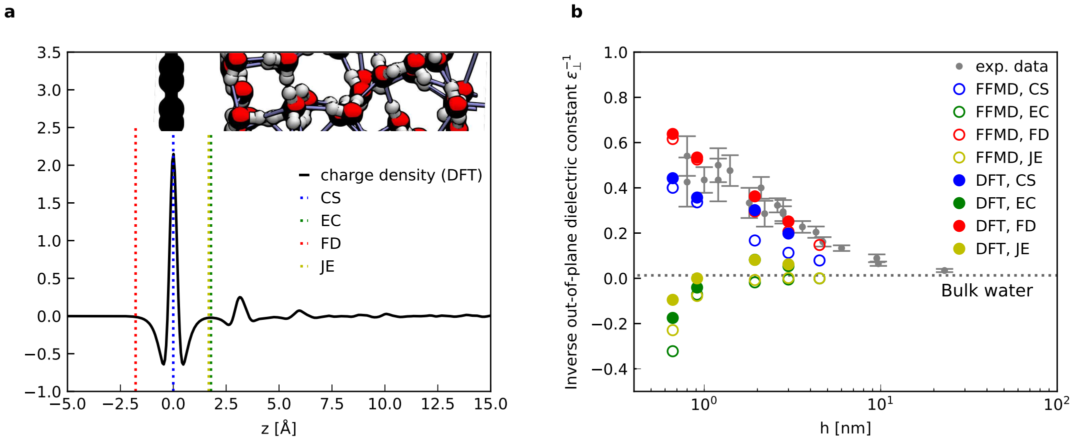

Figure S4: Impact of the dividing surface. (a) Examples of positions of the dividing surface (dashed vertical lines) at the carbon atom side and calculated charge density profile using DFT calculations (solid line). A snapshot of the system is provided as a guidance. (b) Calculated inverse dielectric constant of water for various dividing surfaces shown in (a) using DFT and FFMD calculations.

that shifting the dividing surface to the FD surface would lead to an even better agreement, but it would not reflect the experimental setup. Indeed, it would imply to compute the dielectric constant of the first water layer (L1) over a spatial domain larger than the experimental system, thus artificially reducing the dielectric constant. Importantly, the position of the dividing surface does not affect the calculation of the dielectric constant of the second water layer (L2), or the dipole moments, and the water molecular arrangement presented here. We also note that for all the dividing surfaces considered here, our AIMD calculations yielded smaller $\epsilon_\perp$ than the values obtained from force-field simulations.

S3 Additional force-field simulations

We compared the out-of-plane dielectric constant, $\epsilon_\perp$, of confined water calculated from our AIMD simulations with values obtained from FFMD simulations with the aim of evaluating the performance of our simulations and the impact of different force-fields. Figure S5 shows different FFMD simulations, either reported in the literature or carried out in this study, of water confined in graphene slits as a function of the water slab thickness against the experimental data of Ref.[1]. The water models used in the FFMD simulations reported here from the literature are SPCE [4] (used in [5, 6, 7]) and KKY [8] (used in [9]), a more complex model that allows intramolecular vibrations. Graphite and graphene are modeled by rigid carbon walls using the carbon-water two body interactions. The force-field models are parametrized from various sources, namely, DFT calculations [9] using a Buckingham potential based on [10], properties of water droplets on graphene [6] using a carbon-oxygen van der Waals potential [11], or properties of biomolecules [7] using the GROMOS53A6 force field [12] (a carbon-oxygen Lennard-Jones interaction) or a Lennard-Jones potential using varying parameters in [5]. In the FFMD simulations carried out in this study, we used three different water-carbon interaction potentials, with two 2-body potentials, one adjusted on DFT which was used for the data reported in the main text [13] and one on [14], and a carbon wall potential adjusted on water adsorption from quantum Monte Carlo data [15]. Figure S5 clearly shows that our simulations gives similar results as reported in literature, with all FFMD simulations yielding a similar decrease of $\epsilon_\perp$ as a function of the water slab thickness, irrespective of the force field used. In particular, they all match for water under strong confinement, when the distance between the confining surface is less than 1 nm. This indicates that when the strength of the confinement becomes large, the exact details of the interaction have no impact. Indeed, under strong confinement, the orientation of the water molecule is constrained in the in-plane direction due to the small space available in the slit, thus the ability of the water dipole to reorient with the external electric field is strongly suppressed. For larger water slabs, the shift in the predicted $\epsilon_\perp$ associated with different force fields remains substantially negligible.

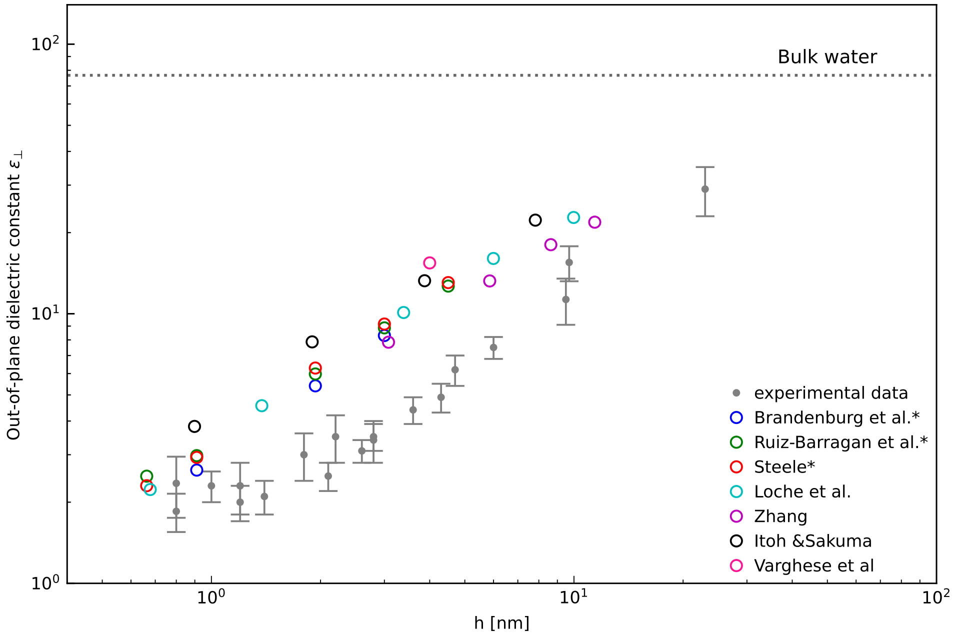

Figure S5: Impact of the force-field choice. Calculated out-of-plane dielectric constant of water confined in graphene slits using different force-fields in literature, reported in the following references: Brandenburg *et al.* [16], Ruiz-Barragan *et al.* [13], Steele [14], Loche *et al.* [7], Zhang [5], Itoh & Sakuma [9], Varghese *et al.* [6]. Symbols labeled with a star (*) correspond to simulations made in this study, while the rest of simulated data are reported here from the references. Grey symbols are the experimental data reported here from Ref.[1].

We also investigated the effect of the water density on the calculated dielectric constant of the water confined inside graphene slits, as the water density in the slits used in the experiment is not known. To this aim, we run FFMD simulations with different values of water density ranging from 0.95 to 1.4 g/cm^3, corresponding to lateral pressures between -0.3 kbar and around 6 kbar inside the confined region. Results are plotted in Fig. S6, again against the experimental data, clearly showing that the change in water density range considered here has only a minor impact on the predicted values of $\epsilon_\perp$ of confined water. The small variation in $\epsilon_\perp$ observed here arises from the competition

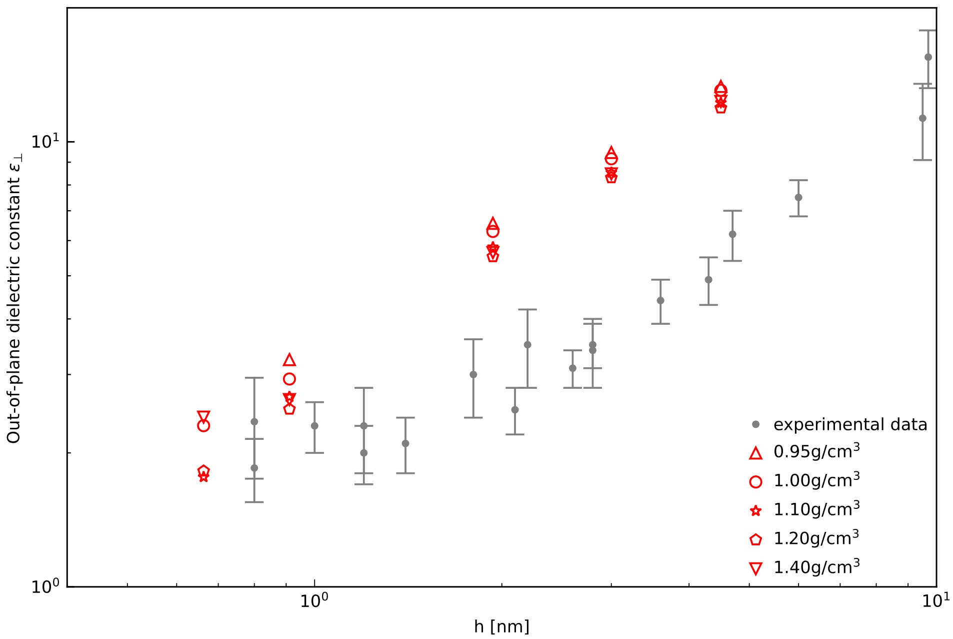

Figure S6: Impact of bulk water density on the dielectric constant of confined water. Calculated dielectric constant of water confined in graphene slits for different values of bulk water density. Grey symbols are the experimental data reported here from Ref.[1].

between two effects. On the one hand, increasing the water density increases the number of molecular dipoles per volume unit. If the response of individual dipole moments remains unchanged, the polarization response is then expected to increase. On the other, increasing the water density would increase the rigidity of the hydrogen bonding network, which in turn decreases the ability of the water dipoles to reorient with the external electric field. Our simulations show a small decrease in $\epsilon_\perp$ with increasing the water density up to 1.1 g/cm^3, suggesting the increased connectivity of the hydrogen bonding network is dominant at relatively low density values. For density larger than 1.1 g/cm^3, the trend reverses, indicating a small increase in $\epsilon_\perp$, thus the increase of dipole density becomes the dominant effect. However, the variations obtained here clearly show that both effects are not significant, with a decrease in $\epsilon_\perp$ of only 10% for a change in water density of 20%.

S4 Hydrogen bond orientation

For the analysis of the hydrogen bonding network inside the slit described in the main text, we used the hydrogen bond definition based on a distance criterion between the two oxygen atoms r_{OO} and an angle criterion on the $\theta_{\mathrm{HO}_d\mathrm{O}_a}$ angle, where O_d is the oxygen of the donor molecule and O_a is the oxygen of the acceptor [17]. The two molecules form a hydrogen bond if $r_{\mathrm{OO}} \leq 3.5$ Å, and $\theta_{\mathrm{HO}_d\mathrm{O}_a} \leq 30°$. The direction of the hydrogen bond is then defined as the direction of the $\overrightarrow{\mathrm{O}_d\mathrm{O}_a}$ vector.

Figure S7 compares the H-bond distributions obtained from NNP simulations, reported in Fig. 3 in the main text, with the distributions obtained from our FFMD simulations in the first two interfacial water layers near the graphene interface. From the comparison between the two data set, it can be seen that the obtained orientation is similar, but the NNP simulations yield a larger proportion of out-of-plane hydrogen bonds and in a smaller angular window in the first interfacial layer (L1). This indicates that a more rigid hydrogen-bonding network is predicted by NNP simulations as opposed to FFMD simulations.

Table S3 compares the average number of hydrogen bonds for the interfacial layers of water confined by carbon and hBN from NNP simulations. As observed already in FFMD simulations [18], the average number of hydrogen bonds is a non-monotonic function of the slit size, reaching a maximum for the bilayer case. This suggests a high structural order of water molecules in the bilayer. We note that the average number of hydrogen bonds for the interfacial layer L1 is found to be lower by 10% compared to what was reported for FFMD simulations [18]. We also observed a significant drop in the number of hydrogen bonds when going from bilayer to monolayer, even larger than what observed in FFMD simulations [18].

Table S4 compares the average dipole moment of the water molecules in the interfacial layers confined by carbon and hBN from NNP simulations. We note that these results point towards a correlation between the average number of hydrogen bonds per molecule, and thus the local structure of water and the average dipole moment as seen for bulk water [19], which was expected from weakening of covalent bonds upon hydrogen-bond formation.

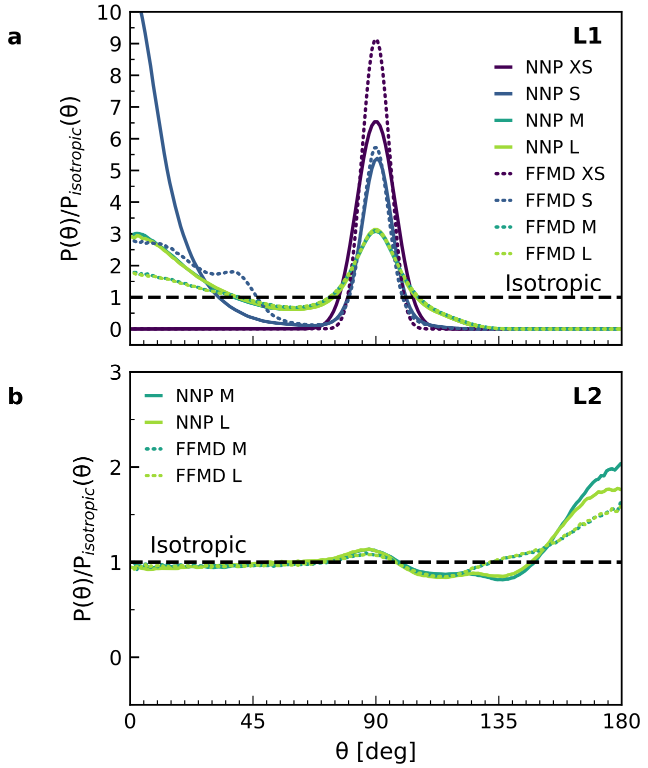

Figure S7: Orientation of hydrogen bonds in the first two interfacial layers. Orientation distribution with respect to the normal direction of the hydrogen bonds for water (a) in the first and (b) the second interfacial layer (L1 and L2) near the graphene surface, normalized by the isotropic distribution, calculated using NNP (solid lines) and FFMD (dashed lines) simulations for various slab thicknesses. The direction corresponds to the donor oxygen-acceptor oxygen.

Table S3: Average number of water hydrogen bonds in the first two interfacial layers. Number of the hydrogen bonds of the interfacial water layers molecules, L1 and L2, reported in Fig. 1b in the main text, for both graphene (first column) and hBN slits (second column) and various water slab thickness using AIMD simulations.

h (nm)	n_H^C	n_H^{hBN}
	L1	
3.000	2.93 ± 0.19	2.94 ± 0.19
1.935	2.98 ± 0.18	2.87 ± 0.18
0.911	3.09 ± 0.21	3.12 ± 0.21
0.663	2.19 ± 0.25	2.18 ± 0.25
	L2	
3.000	3.49 ± 0.16	3.47 ± 0.17
1.935	3.54 ± 0.15	3.43 ± 0.16

Table S4: Average molecular dipole moment of water in the first two interfacial layers. Calculated molecular dipole of the water molecules in the interfacial layers L1 and L2, reported in Fig. 1b in the main text, for both graphene (first column) and hBN slits (second column) and various water slab thickness using AIMD simulations.

h (nm)	μ_{H2O}^C (D)	μ_{H2O}^{hBN} (D)
	L1	
3.000	2.744 ± 0.053	2.740 ± 0.056
1.935	2.755 ± 0.050	2.724 ± 0.053
0.911	2.770 ± 0.057	2.769 ± 0.059
0.663	2.603 ± 0.070	2.601 ± 0.070
	L2	
3.000	2.869 ± 0.052	2.868 ± 0.052
1.935	2.885 ± 0.052	2.851 ± 0.051

S5 Influence of DFT Functional

In this subsection, we investigated how the main structural properties of interfacial water change with the chosen DFT functional. To this end, we used two separate NNP models trained to the PBE0 functional and the improved dispersion correction D4 as described in detail in Ref.[20]. This hybrid functional was shown to perform very well in comparison to higher level benchmarks of the water-carbon interactions [16]. Based on these MLPs we performed additional simulations of the L [3.00 nm] system for the two confining materials with otherwise identical settings as before. A detailed comparison of the structural properties of water for both the revPBE-D3 functional, used in the main text, and the hybrid functional PBE0-D4 is shown in Fig. S8. While there are minor differences in the density profile, the dipole orientation and hydrogen bonding orientation in the first two interfacial layers is in good agreement between the two functionals. The agreement is best for the second layer, L2, while PBE0-D4 predicts slightly less structured molecular orientations in the first layer, L1. Nevertheless, the main findings reported in the main text remain unchanged for this functional, as similarly broad antiparallel dipole arrangements are seen for PBE0-D4. Most importantly, PBE0-D4 also predicts very different dipole orientations compared to FFMD. We thus conclude that the choice of DFT functional does not significantly impact the interfacial structural properties of water when using well performing functionals with respect to higher level benchmarks for the material-water interactions.

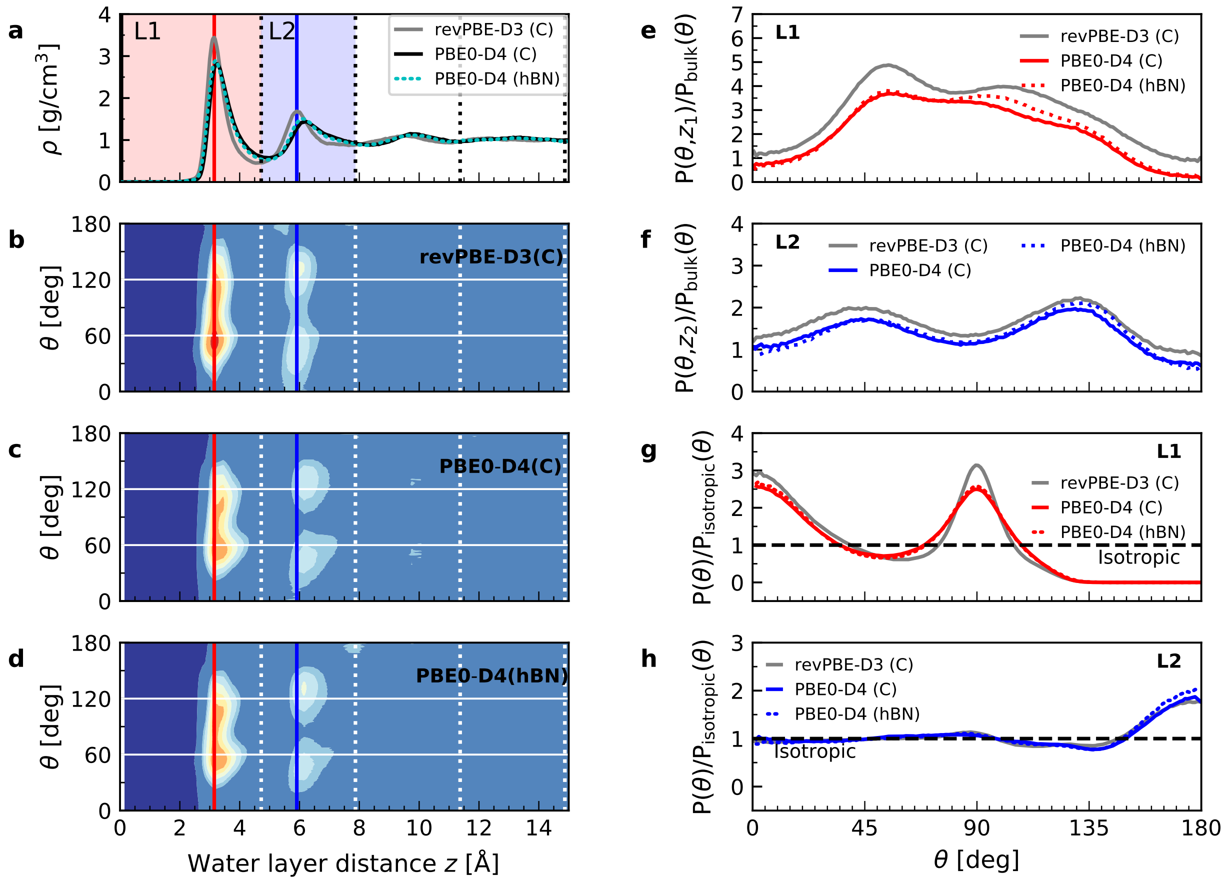

Figure S8: Influence of DFT functional on properties of interfacial water. **(a)** Mass density profile obtained from NNP simulations with the revPBE-D3 functional for graphene (gray solid line), as well as the PBE0-D4 functional for graphene (black solid line) and hBN (green dashed line) for h=3.0 nm [M]. **(b)** Distribution of the water dipole moment angle with respect to the perpendicular direction as a function of the distance from the surface for graphene obtained with the revPBE-D3 NNP. **(c,d)** Same as **(b)** but using PBE0-D4 NNP simulations for **(c)** graphene and **(d)** hBN. **(e,f)** Dipole orientation distribution profiles at distance **(e)** $z = 3$ Å (L1) and **(g)** $z = 6$ Å (L2), corresponding to the red and blue vertical dashed lines in **(b,c,d)**, for revPBE-D3 NNP simulations of graphene and PBE0-D4 NNP simulations for graphene as well as hBN. The distributions are normalized by the isotropic distribution of bulk water with density of 1 gcm^{-3}. **g,h** Calculated orientation distribution with respect to the normal direction of the hydrogen bonds from revPBE-D3 NNP simulations of water on graphene and PBE0-D4 NNP simulations of water on graphene and hBN in the first **(g)** and second **(h)** interfacial layers, normalized by the isotropic distribution.

S6 Influence of Nuclear Quantum Effects

Given the small mass of the hydrogen atoms, some properties of water can depend significantly on the quantum nature of the nuclei. We performed additional thermostatted ring polymer dynamics simulations [21] to investigate if these nuclear quantum effects (NQEs) have an impact on the structural properties of interfacial water, as reported in the main text. These simulations were performed with the CP2K[22] simulation package. Using the L [3.00 nm] system for both confining materials, each atom was represented by 16 replica to achieve convergence of structural properties. The timestep was reduced to 0.25 fs, while using a path integral Langevin equation thermostat [23] for the non-centroid modes. Due to the higher computational costs, we restricted the simulations to 500 ps for both confinement materials. These simulations provide access to converged quantum statistical properties and allow us to directly compare to simulations with classical nuclei that do not account for NQEs. As shown in Fig. S9, all relevant structural properties of interfacial water reported in the main text are essentially identical for classical and quantum nuclei. This includes the density profile, molecular orientation, and hydrogen bond orientation in the first two contact layers. From this analysis, we conclude that NQEs to not play an important role in the interfacial properties of water under confinement.

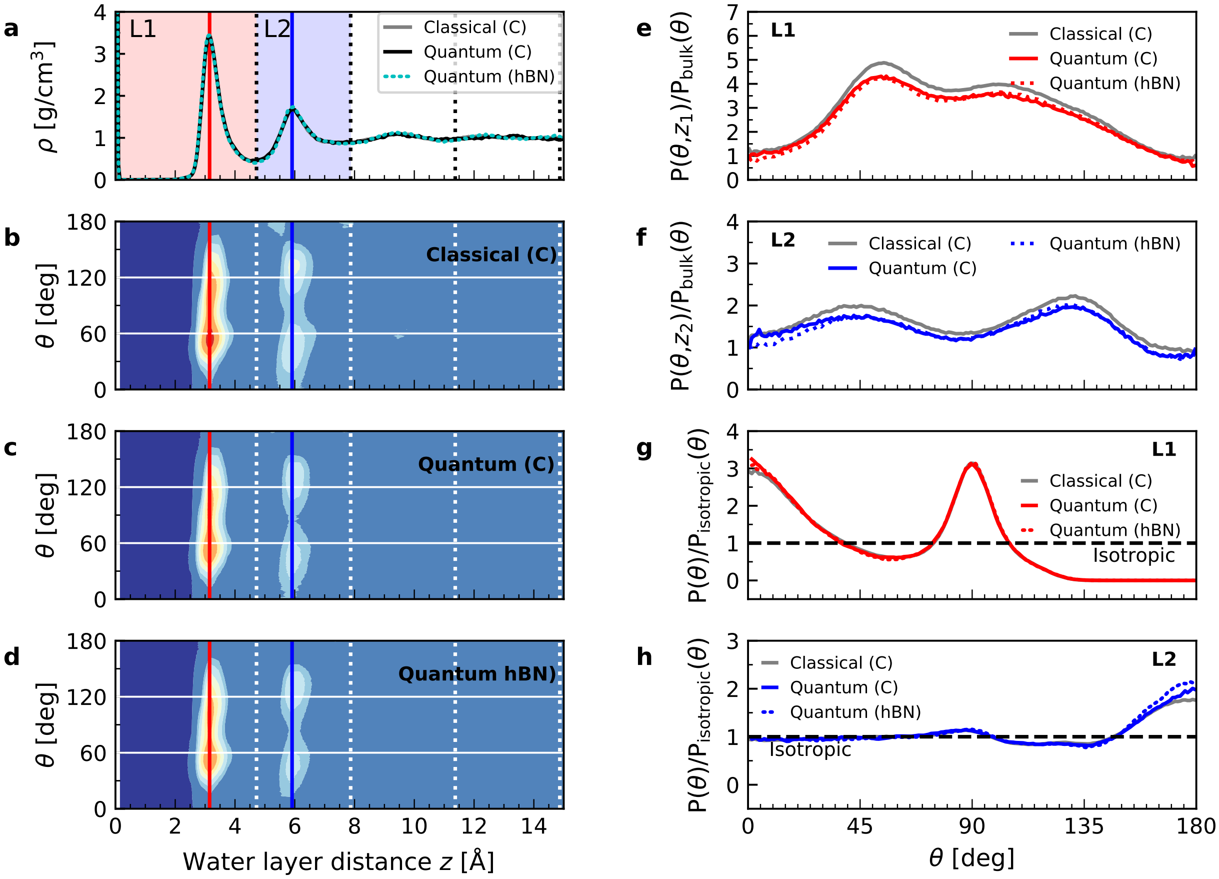

Figure S9: Influence of nuclear quantum effects on properties of interfacial water. **(a)** Mass density profile obtained from NNP simulations for graphene with classical nuclei (gray solid line), as well as the quantum nuclei for graphene (black solid line) and hBN (green dashed line) for h=3.0 nm [M]. **(b)** Distribution of the water dipole moment angle with respect to the perpendicular direction as a function of the distance from the surface for graphene obtained with classical nuclei. **(c,d)** Same as **(b)** but using quantum nuclei for **(c)** graphene and **(d)** hBN. **(e,f)** Dipole orientation distribution profiles at distance **(e)** $z = 3$ Å (L1) and **(g)** $z = 6$ Å (L2), corresponding to the red and blue vertical dashed lines in **(b,c,d)**, for classical nuclei of water on graphene and quantum nuclei for water on graphene as well as hBN. The distributions are normalized by the isotropic distribution of bulk water with density of 1 gcm^{-3}. **g,h** Calculated orientation distribution with respect to the normal direction of the hydrogen bonds for classical nuclei of water on graphene and quantum nuclei of water on graphene and hBN in the first **(g)** and second **(h)** interfacial layers, normalized by the isotropic distribution.

References

[1] Fumagalli, L. *et al.* Anomalously low dielectric constant of confined water. *Science* **360**, 1339–1342 (2018).

[2] Zhang, C. & Sprik, M. Computing the dielectric constant of liquid water at constant dielectric displacement. *Phys. Rev. B* **93**, 144201 (2016).

[3] Smith, N., Chen, C. & Weinert, M. Distance of the image plane from metal surfaces. *Phys. Rev. B* **40**, 7565 (1989).

[4] Berendsen, H., Grigera, J. & Straatsma, T. The missing term in effective pair potentials. *J. Phys. Chem.* **91**, 6269–6271 (1987).

[5] Zhang, C. Note: On the dielectric constant of nanoconfined water. *J. Chem. Phys.* **148**, 156101 (2018).

[6] Varghese, S., Kannam, S., Hansen, J. & P. Sathian, S. Effect of hydrogen bonds on the dielectric properties of interfacial water. *Langmuir* **35**, 8159–8166 (2019).

[7] Loche, P., Ayaz, C., Wolde-Kidan, A., Schlaich, A. & Netz, R. Universal and nonuniversal aspects of electrostatics in aqueous nanoconfinement. *J. Phys. Chem. B* **124**, 4365–4371 (2020).

[8] Kumagai, N., Kawamura, K. & Yokokawa, T. An interatomic potential model for H_2O: applications to water and ice polymorphs. *Mol. Simul.* **12**, 177–186 (1994).

[9] Itoh, H. & Sakuma, H. Dielectric constant of water as a function of separation in a slab geometry: A molecular dynamics study. *J. Chem. Phys.* **142**, 184703 (2015).

[10] Cortes, E., Solís, L. & Arellano, J. Interaction of a water molecule with a graphene layer. *Rev. Mex. de Fis.* **59**, 118–125 (2013).

[11] Werder, T., Walther, J., Jaffe, R., Halicioglu, T. & Koumoutsakos, P. On the water-carbon interaction for use in molecular dynamics simulations of graphite and carbon nanotube. *J. Phys. Chem.* **107**, 1345–1352 (2003).

[12] Oostenbrink, C., Villa, A., Mark, A. & Van Gunsteren, W. A biomolecular force field based on the free enthalpy of hydration and solvation: the GROMOS force-field parameter sets 53A5 and 53A6. *J. Comput. Chem.* **25**, 1656–1676 (2004).

[13] Ruiz-Barragan, S., Muñoz-Santiburcio, D., Körning, S. & Marx, D. Quantifying anisotropic dielectric response properties of nanoconfined water within graphene slit pores. *Phys. Chem. Chem. Phys.* **22**, 10833–10837 (2020).

[14] Steele, W. *The interaction of gases with solid surfaces*, vol. 3 (Pergamon, 1974).

[15] Brandenburg, J. *et al.* Physisorption of water on graphene: subchemical accuracy from many-body electronic structure methods. *J. Phys. Chem. Lett.* **10**, 358–368 (2019).

[16] Brandenburg, J., Zen, A., Alfe, D. & Michaelides, A. Interaction between water and carbon nanostructures: How good are current density functional approximations? *J. Chem. Phys.* **151**, 164702 (2019).

[17] Marti, J. Analysis of the hydrogen bonding and vibrational spectra of supercritical model water by molecular dynamics simulations. *J. Chem. Phys.* **110**, 6876–6886 (1999).

[18] Leoni, F., Calero, C. & Franzese, G. Nanoconfined fluids: Uniqueness of water compared to other liquids. *ACS nano* **15**, 19864–19876 (2021).

[19] Skarmoutsos, I., Franzese, G. & Guardia, E. Using car-parrinello simulations and microscopic order descriptors to reveal two locally favored structures with distinct molecular dipole moments and dynamics in ambient liquid water. *J. Mol. Liq.* **364**, 119936 (2022).

[20] Thiemann, F. L., Schran, C., Rowe, P., Müller, E. A. & Michaelides, A. Water flow in single-wall nanotubes: Oxygen makes it slip, hydrogen makes it stick. *ACS Nano* **16**, 10775–10782 (2022).

[21] Rossi, M., Ceriotti, M. & Manolopoulos, D. E. How to remove the spurious resonances from ring polymer molecular dynamics. *J. Chem. Phys.* **140**, 234116 (2014).

[22] Kühne, T. D. *et al.* CP2K: An electronic structure and molecular dynamics software package - quickstep: Efficient and accurate electronic structure calculations. *J. Chem. Phys.* **152**, 194103 (2020).

[23] Ceriotti, M., Parrinello, M., Markland, T. E. & Manolopoulos, D. E. Efficient stochastic thermostatting of path integral molecular dynamics. *J. Chem. Phys.* **133**, 124104 (2010).
